# TSPAN8 promotes cancer cell stemness via activation of sonic Hedgehog signaling

**DOI:** 10.1038/s41467-019-10739-3

**Published:** 2019-06-28

**Authors:** Rongxuan Zhu, Olivier Gires, Liqun Zhu, Jun Liu, Junjian Li, Hao Yang, Gaoda Ju, Jing Huang, Weiyu Ge, Yi Chen, Zhimin Lu, Hongxia Wang

**Affiliations:** 1 Department of Oncology, Shanghai General Hospital, Shanghai Jiao Tong University School of Medicine, Shanghai, 200080 China; 2 Department of Otorhinolaryngology, Head and Neck Surgery, Grosshadern Medical Center, Ludwig-Maximilians-University of Munich, Munich, Germany; 3 Liyang People’s Hospital, Liyang, Jiangsu 213300 China; 4 Department of Breast-Thyroid-Vascular Surgery, Shanghai General Hospital, Shanghai Jiao Tong University School of Medicine, Shanghai, 201620 China; 5 Shanghai Key Laboratory of Molecular Imaging, Shanghai University of Medicine and Health Sciences, Shanghai, 201318 China; 6 School of Pharmaceutical Science and Technology, Dalian University of Technology, Dalian, 116024 China; 7 Division of Anti-Tumor Pharmacology, State Key Laboratory of Drug Research, Shanghai Institute of Materia Medica, Chinese Academy of Sciences, Shanghai, 201203 China; 8 Zhejiang Provincial Key Laboratory of Pancreatic Disease, The First Affiliated Hospital, and Institute of Translational Medicine, Zhejiang University School of Medicine, Hangzhou, 310029 China

**Keywords:** Breast cancer, Cancer stem cells

## Abstract

Cancer stem cells (CSCs) represent a major source of treatment resistance and tumor progression. However, regulation of CSCs stemness is not entirely understood. Here, we report that TSPAN8 expression is upregulated in breast CSCs, promotes the expression of the stemness gene NANOG, OCT4, and ALDHA1, and correlates with therapeutic resistance. Mechanistically, TSPAN8 interacts with PTCH1 and inhibits the degradation of the SHH/PTCH1 complex through recruitment of deubiquitinating enzyme ATXN3. This results in the translocation of SMO to cilia, downstream gene expression, resistance of CSCs to chemotherapeutic agents, and enhances tumor formation in mice. Accordingly, expression levels of TSPAN8, PTCH1, SHH, and ATXN3 are positively correlated in human breast cancer specimens, and high TSPAN8 and ATXN3 expression levels correlate with poor prognosis. These findings reveal a molecular basis of TSPAN8-enhanced Sonic Hedgehog signaling and highlight a role for TSPAN8 in promoting cancer stemness.

## Abstract (web summary)

Tetraspanin 8 (TSPAN8) has been implicated in a number of different tumours, but the underlying mechanisms remain unclear. Here, in breast cancer the authors highlight a role for TSPAN8 in promoting tumorigenesis through the activation of Hedgehog signalling.

## Introduction

Cancers represent a collection of highly heterogeneous malignant diseases that comprise cellular hierarchies defined as sub-populations of self-renewing cancer stem cell (CSCs) within a majority of more differentiated cancer cells. CSCs are regarded as the major source of resistance toward conventional therapeutic regimens and the roots of metastasis and recurrence^1–8^. Despite the central role of CSCs in cancer progression, the regulation of their key features, such as stemness and tumorigenicity, remains incompletely understood.

The Hedgehog (Hh) signaling is an evolutionarily conserved pathway, which is essential for cell fate determination and self-renewal. It is reported that aberrant Hh signaling is associated with development and progression of multiple types of cancer and is involved in the maintenance of CSCs^9–11^. Hh signaling is activated by the binding with Hh ligands, such as Desert Hedgehog (Dhh), Indian Hedgehog (Ihh), and Sonic Hedgehog (Shh), to their cognate receptors, Patched (Ptch1 as well as, to a lesser extent, Ptch2). The binding of ligand relieves the suppression of Smoothened (Smo) through PKA-, CK1α-, or G-protein-coupled receptor kinase-2 (Grk2)-mediated phosphorylation of Smo, triggering an interaction of Smo with Kif3a and β-arrestin (Arrb2), and subsequent ciliary localization of Smo^12^. These series of processes facilitate the release of full-length transcriptionally active Gli proteins (GliA) from the suppressor of fused (Sufu). Then GliA translocates into the nucleus to activate the Hh-targeted genes, such as the Hh pathway genes, *Ptch1*, *Gli1*, and *hedgehog-interacting protein* (*Hhip*), thereby forming feedback loops that reduce or enhance the Hh response. Simultaneously, Ptch1 is internalized and degraded within lysosomes. CSCs respond to Hh ligands by maintaining a stemness signature through the influences on pluripotency genes, including *Sox2*, *Nanog*, and *Bmi1*^13–15^. In spite of the importance of Hh signaling in cancer development, the mechanism underlying the regulation of Ptch1 receptors in response to Hh binding remains to be elucidated.

Tetraspanins are membrane glycoproteins constituting a family of 33 members in mammals, including tetraspanin-8 (TSPAN8 encoded by *TSPAN8* gene) and several clusters of differentiation (CD) related proteins, such as CD63, CD37, CD53, CD81, and CD9^16^. The name-giving common feature of tetraspanins is the four highly conserved membrane-spanning domains. Generally, tetraspanins play major roles in a plethora of cellular functions. Increasing evidence suggests that TSPAN8 promotes tumor cell migration, invasion, and metastasis in multiple types of human cancers, including ovarian and gastric colorectal cancers, hepatocarcinoma, pancreatic adenocarcinoma, and glioma^17–20^. However, the mechanisms underlying the role of TSPAN8 in the regulation of tumor progression remain largely unknown.

In the study, we demonstrate TSPAN8 interacts with SHH-PTCH1 complex and enhances the binding of PTCH1 to SHH and the release of SMO from PTCH1. In addition, TSPAN8 recruits ATXN3 deubiquitinating enzyme to reduce ubiquitination of PTCH1 and inhibits the proteasome-mediated degradation of the SHH/PTCH1 complex. Stabilized SHH/PTCH1 promotes the binding of GRK2 protein kinase to SMO and the subsequent SMO phosphorylation, translocation of SMO to cilia, and GLI1 activation for downstream gene expression.

## Results

### TSPAN8 expression is upregulated in breast CSCs

**Fig. 1:**
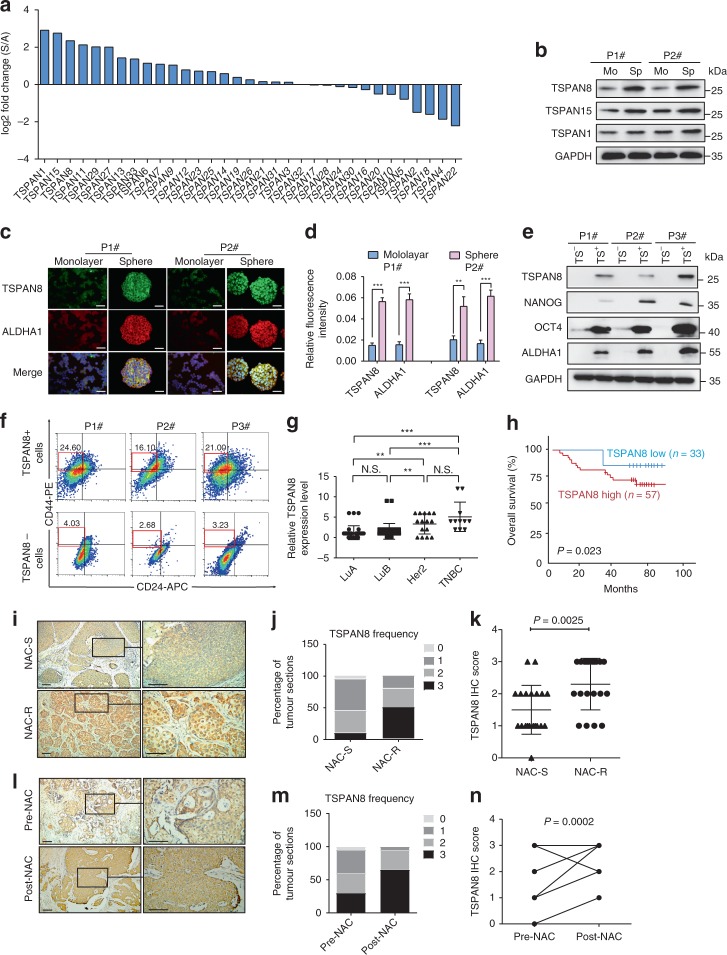
TSPAN8 expression is upregulated in breast CSCs. **a** Microarray analyses of expression of 33 members of tetraspanins family expressed in CSCs (spheres) or their corresponding non-CSCs (adherent cells) derived from three independent breast cancer patients. **b** Freshly isolated primary human tissues derived from two independent breast cancer patients were grown as adherent cells or spheres. Immunoblotting analyses of TSPAN8, TSPAN15 and TSPAN1 were performed. **c** Representative confocal images for TSPAN8 (green), ALDHA1 (red), and nuclei (blue) of adherent cells or spheres from two breast cancer patients were shown. **d** Quantification of the relative fluorescence intensity of TSPAN8 and ALDHA1 in spheres or adherent cells. Two-tailed Student’s *t* test was used for statistical analysis. ***P < 0.001, **P < 0.01, *P < 0.05. **e** Immunoblotting analyses of TSPAN8, NANOG, OCT4, and ALDHA1 expression in breast cancer cells from three patients were performed. TS^−^: TSPAN8-negative breast cancer cells, TS^+^: TSPAN8-positive breast cancer cells. **f** Flow cytometry analysis of the ratios of CD44^+^/CD24^−^ cells in TS^+^ breast cancer cells or TS^−^ breast cancer cells derived from three human breast cancer patients. **g** Semi-quantitative analyses of IHC staining of TSPAN8 in tissue sections of 90 breast cancer patients with different pathological molecular subtypes. ***P < 0.001, **P < 0.01, *P < 0.05 and N.S., not significant (*P* > 0.05) by repeated measures with Student’s *t* test. LuA = luminal A subtype, LuB = luminal B subtype, Her2 = Her2 amplified subtype, TNCB = triple-negative subtype. **h** Kaplan–Meier of survival of 90 patients with breast tumors (two groups stratified by TSPAN8 expression level. Differences between the groups were shown by a log-rank test. **i**–**k** Immunohistochemistry analyses of TSPAN8 expression in specimens of breast cancer patients with NAC-S (neo-adjuvant chemotherapy sensitive) and NAC-R (neo-adjuvant chemotherapy resistant) characteristics (scale bar = 50 μm, *n* = 20). **l**–**n** Immunohistochemistry analyses of TSPAN8 expression in specimens of breast cancer patients with Pre-NAC (before neo-adjuvant chemotherapy) and Post-NAC (after neo-adjuvant chemotherapy) (scale bar = 50 μm, *n* = 20). Note that some dots overlapped

To identify key regulators of CSCs stemness, we carried microarray analyses of primary breast cancer spheres derived from breast cancer patients and the corresponding cultured adherent cells (referred to as non-CSCs hereafter). As expected, breast cancer spheres expressed a profile of genes, which were similar to reported CSCs gene signatures^21^ (Supplementary Fig. 1a). Analyses of the expression levels of all 33 tetraspanins revealed significantly higher expression of *TSPAN1, TSPAN15, TSPAN8, TSPAN11, TSPAN29*, and *TSPAN27* in the breast cancer spheres than in non-CSCs (Fig. 1a). We found the protein level of TSPAN8, which is correlated with cancer progression, was strongly upregulated in the breast cancer spheres (Fig. 1b). This result was further confirmed by immunofluorescent analyses, which showed that TSPAN8 and ALDHA1, a functional marker of progenitor and cancer stem cells^22^, were overexpressed in breast cancer spheres (Fig. 1c, d). To further determine whether TSPAN8 is a CSCs marker, we used flow cytometry to separate TSPAN8-highly expressed (TS^+^) from TSPAN8-lowly expressed (TS^−^) cells in non-cultured primary breast cancer cells derived from three independent patients. We showed that expression of NANOG and OCT4, which are transcription factors involved in the maintenance of the pluripotent state of stem cells^23,24^, was enhanced in the TS^+^ cells (Fig. 1e). Similarly, real-time PCR (Supplementary Fig. 1b, c, and d) and immunoblotting analyses (Supplementary Fig. 1e) revealed that the transcription and protein expression levels of TSPAN8, NANOG, SOX2, as well as ALDHA1 were significantly higher in spheres derived from MCF7, HCC1954, and MDA-MB-231 breast cancer cells than those in the corresponding adherent cells. Replacement of the stem cell culture medium with adherent culture medium reduced the expression of these genes, suggesting that the expression of TSPAN8, NANOG, SOX2, and ALDHA1 is induced in spheres. In addition, *TSPAN8* overexpression in MCF7 cells significantly enhanced both the mRNA and protein expression levels of SOX2, OCT4, NANOG, and ALDHA1 (Supplementary Fig. 1f, g). In contrast, a decrease of these expression levels was observed by expressing *TSPAN8*-specific short hairpin RNA (shRNA) (Supplementary Fig. 1h, i). These results indicated that TSPAN8 expression is associated with cancer stem cell phenotypes.

Compared to 3.73 ± 0.24% of CD44-positive (CD44^+^)/CD24-negative (CD24^−^) cells in EpCAM-positive (EpCAM^+^)/CD45^−^ negative (CD45^−^)/CK-positive (CK^+^) breast cancer cells isolated from 10 breast cancer patients, the percentage of TS^+^ cells in total breast cancer cells were 22.51 ± 0.92%, which was higher than that in non-tumor cells from adjacent breast tissues (Supplementary Fig. 1j). Analyses of the relationship between CD44^+^/CD24^−^ and TS^+^ in breast cancer cells, we observed a substantially higher percentage of CD44^+^/CD24^−^ cells in TS^+^ cells than in TS^−^ cells (Fig. 1f). In addition, 66.45 ± 1.63% of CD44^+^/CD24^−^ subgroup of breast cancer cells were TS^+^ cells (Supplementary Fig. 1k). These resulted further supported that TS^+^ cells represent a stem cell population.

We next examined TSPAN8 expression levels in patients with different molecular pathological breast cancer subtypes, including luminal A, luminal B, HER2, and triple-negative breast cancer (TNBC). The microarray analysis of tumor samples of 90 breast cancer patients showed that TSPAN8 was highly expressed in TNBC and lowly expressed in luminal subtype (Fig. 1g). Importantly, TSPAN8 expression levels were reversely correlated with the overall survival time of breast cancer patients (Fig. 1h).

To further investigate the clinical significance of TSPAN8 expression, we performed immunohistochemical staining (IHC) analyses of an additional cohort of human breast cancer specimens, determined the correlation between TSPAN8 expression and the response rate to chemotherapy. All patients recruited in the study received neo-adjuvant chemotherapy (NAC) and were stratified into a NAC-sensitive group (*n* = 20) and a NAC-resistant group (*n* = 20). The clinical curative effect of primary breast cancer was evaluated based on RECIST 1.1 (Response Evaluation Criteria in Solid Tumors) measurement criteria^25^. The expression of TSPAN8 was evidently higher in the NAC-resistant group than in the NAC-sensitive group (Fig. 1i–1k, *p* = 0.025). Of note, NAC increased the expression of TSPAN8 in treatment-resistant patients (Fig. 1l–n). These results suggested that TSPAN8 expression positively correlates with poor prognosis of breast cancer patients and their therapeutic resistance.

### Overexpression of TSPAN8 enhances characteristics of CSCs

**Fig. 2:**
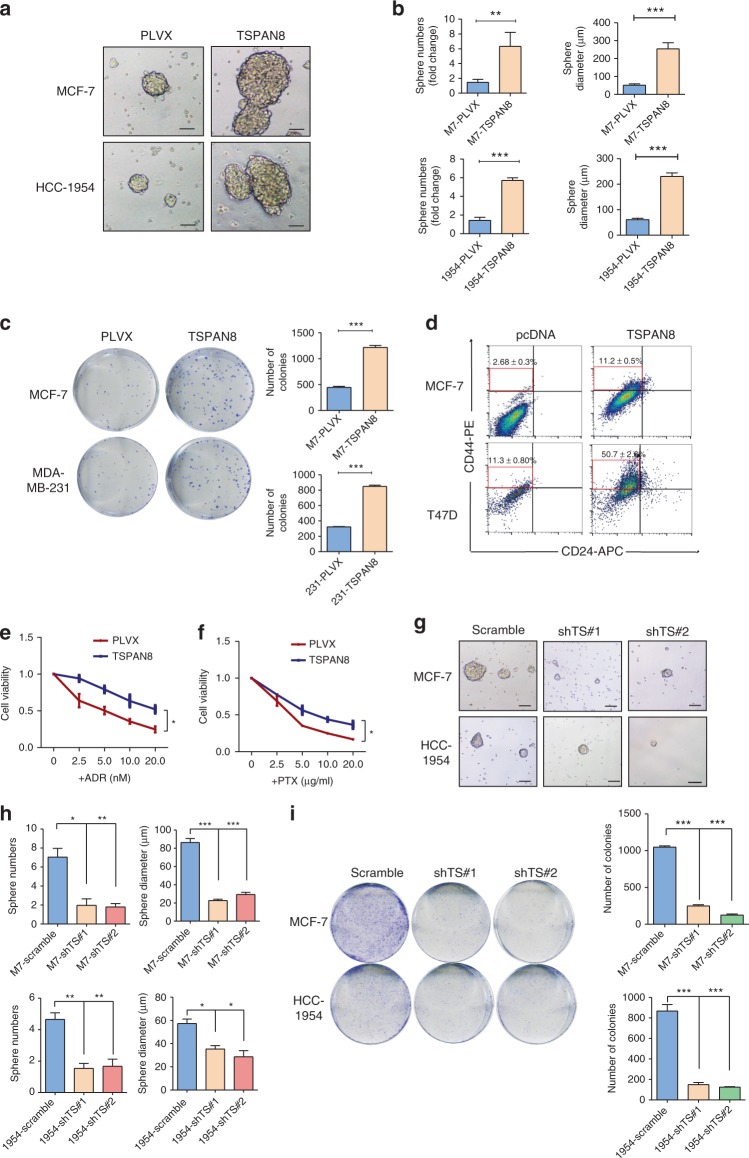
Overexpression of TSPAN8 enhances characteristics of CSCs. **a**, **b** Tumor spheres of representative images of the indicated cells with or without *TSPAN8* overexpression are shown (**a**). Scale bar = 100 μm. Histograms show the mean numbers and diameters of spheres cultured (**b**). Two different cell lines (MCF7, HCC-1954) were used. *P < 0.05, **P < 0.01, and ***P < 0.001 and *n* = 3 biologically independent samples per group. PLVX, lentiviral stable transfection plasmid pLVX-HA-IRES-Puro as the control group. **c** Colony formation assay of the indicated cells with or without *TSPAN8* overexpression was performed. Indicated cells were seeded with 10^3^ numbers per well in a 6-well dish for 3 weeks (*n* = 3). *P < 0.05, **P < 0.01, and ***P < 0.001 and error bars represent standard deviation from three times of independent experiments. **d** Flow cytometry analyses of the ratios with CD44^+^/CD24^−^ cells in the indicated cells with or without *TSPAN8* overexpression were performed. Error bars represent standard deviation from three times of independent experiments. **e**, **f** Parental or *TSPAN8*-stably expressing MCF7 cells were treated with ADR (**e**) or PTX (**f**) at the indicated concentrations for 24 h. Then cell numbers were counted. *P < 0.05, **P < 0.01, and ***P < 0.001 and error bars represent standard deviation from three times of independent experiments. **g**, **h** Tumor sphere forming ability of the indicated cells with or without *TSPAN8* depletion was examined (**g**). Histograms show the mean numbers and diameters of spheres cultured (**h**). Spheres were cultured for a week before counting. Two-tailed unpaired Student’s *t* test was performed. The results were presented as means ± SD from three times of independent experiments. *P < 0.05, **P < 0.01, and ***P < 0.001. Scale bar = 100 µm. **i** Soft agar colony formation assay of the indicated cells with or without *TSPAN8* depletion was performed. Representative images (left) and average number of colonies (right) were shown. The results were presented as means ± SD from three times of independent experiments. Two-tailed unpaired Student’s *t* test was performed. *P < 0.05, **P < 0.01, and ***P < 0.001

*TSPAN8* overexpression increased sphere formation efficiency reflected by the size and numbers of spheres (Fig. 2a–b), colony formation capacity (Fig. 2c), and the number of cells with CD44^+^/CD24^−^ markers (Fig. 2d), and promoted cell growth and survival in the presence of adriamycin (ADR) (Fig. 2e) and paclitaxel (PTX) (Fig. 2f) treatment. In contrast, *TSPAN8* depletion reduced the sphere formation efficiency (Fig. 2g, h), colony formation numbers (Fig. 2i), number of cells with CD44^+^/CD24^−^ markers (Supplementary Fig. 2a), and resistance to chemotherapy (Supplementary Fig. 2b). In line with these results, TS^+^ cells from patient tumor samples exhibited significantly enhanced resistance to paclitaxel (PTX) and Adriamycin (ADR)-induced cytotoxicity (Supplementary Fig. 2c). In addition, FACS (Supplementary Fig. 2d) and immunoblotting (Supplementary Fig. 2e) analyses showed that PTX and ADR increased the ratio of TSPAN8 and enhanced TSPAN8 protein expression in both TS^+^ and TS^−^ primary breast cancer cells. These results reveal that TSPAN8 promotes cancer cell stemness and resistance to chemotherapeutic drug treatment.

### TSPAN8 promotes cancer stemness by activating Sonic Hh

**Fig. 3:**
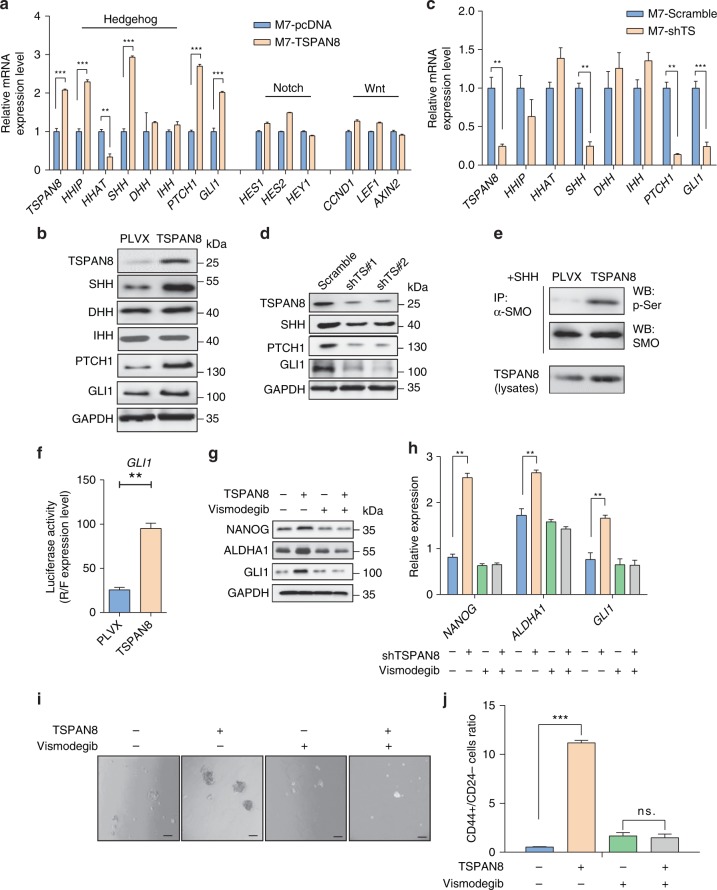
TSPAN8 promotes cells stemness by activating Sonic Hh. **a** qRT-PCR analyses of the mRNA levels of the indicated genes in MCF7 cells with or without *TSPAN8* overexpression (*n* = 3). **b** Immunoblotting analyses of MCF7 cells with or without *TSPAN8* overexpression were performed with the antibodies indicated. GAPDH was used as loading sample control. **c** qRT-PCR analyses of the mRNA levels of the genes indicated in MCF7 cells with or without *TSPAN8* depletion (*n* = 3). Data represent the mean ± SD of three times of independent experiments. ***P < 0.001. **d** Immunoblotting analyses of MCF7 cells with or without *TSPAN8* depletion were performed with the antibodies indicated. GAPDH was used as loading sample control. **e** MCF7 cells with or without *TSPAN8* overexpression were treated with SHH (100 ng/ml) for 6 h. Immunoblotting analyses and immunoprecipitation were performed with the antibodies indicated. P-Ser, phosphorylated serine. **f** Luciferase activities in MCF7 cells infected with lentivirus expressing *TSPAN8* and *GLI1* promoter–luciferase reporter construct were determined. *GLI1* promoter reporter activity was normalized by comparison with Renilla luciferase activity (*n* = 3 per group). Two-tailed unpaired Student’s *t* test was performed. **P < 0.01. **g**–**j** MCF7 cells with or without *TSPAN8* overexpression were treated with or without 10 nM Vismodegib for 6 h and immunoblotting analyses were performed (**g**). qRT-PCR analyses of the mRNA levels were conducted. Data represent the mean ± SD of three times of independent experiments. **P < 0.01 (**h**). Representative images of tumor spheres are shown. Spheres were cultured for a week before counting. Scale bar = 50 µm (**i**). Flow cytometry analyses of the ratios of CD44^+^/CD24^−^ in these cells were performed (**j**)

Wnt, Notch, and Hh signaling pathways are known to play decisive roles in tumor stemness^26^. To assess the potential influence of TSPAN8 on either of these pathways, we constitutively overexpressed *TSPAN8* in MCF7 cells. This overexpression significantly increased the mRNA expression of *PTCH1*, *GLI*, and *SHH* (Hh signaling pathway), but not of *HES1*, *HES2*, and *HEY1* (Notch pathway), or *CCND1*, *LEF1*, and *AXIN2* (Wnt signaling pathway) (Fig. 3a). In line with the results of the mRNA levels, the protein expression levels of PTCH1, GLI, and SHH (not DHH or IHH) were also induced by *TSPAN8* overexpression (Fig. 3b). In contrast, *TSPAN8* depletion reduced the expression of the transcription (Fig. 3c) and protein (Fig. 3d) of these genes, and blocked SHH dosage-dependent upregulation of GLI1 expression (Supplementary Fig. 3a). Accordingly, *TSPAN8* overexpression-enhanced SMO phosphorylation (Fig. 3e) and the transcriptional activity of *GLI1*, as detected by *GLI1*-regulated luciferase reporter analysis (Fig. 3f). In contrast, *TSPAN8* depletion diminished the transcriptional activity of *GLI1* (Supplementary Fig. 3b). Consistently, the treatment with the sonic hedgehog pathway inhibitors Vismodegib and RU-SKI43 decreased the effect of *TSPAN8* overexpression on protein (Fig. 3g, Supplementary Fig. 3c) and mRNA expression (Fig. 3h, Supplementary Fig. 3d) of NANOG, ALDHA1, and GLI1. Furthermore, this treatment reduced the sphere formation efficiency (Fig. 3i, Supplementary Fig. 3e) and the number of CD44^+^/CD24^−^ cancer cells (Fig. 3j, Supplementary Fig. 3f). Of note, *TSPAN8* depletion had limited effect on spheres forming ability of tumor cells treated with Hh inhibitors (Supplementary Fig. 3g). These results suggest that a major feature of TSPAN8 is to regulate Hh signaling.

### TSPAN8 stabilizes the expression of PTCH1 by recruiting ATXN3

**Fig. 4:**
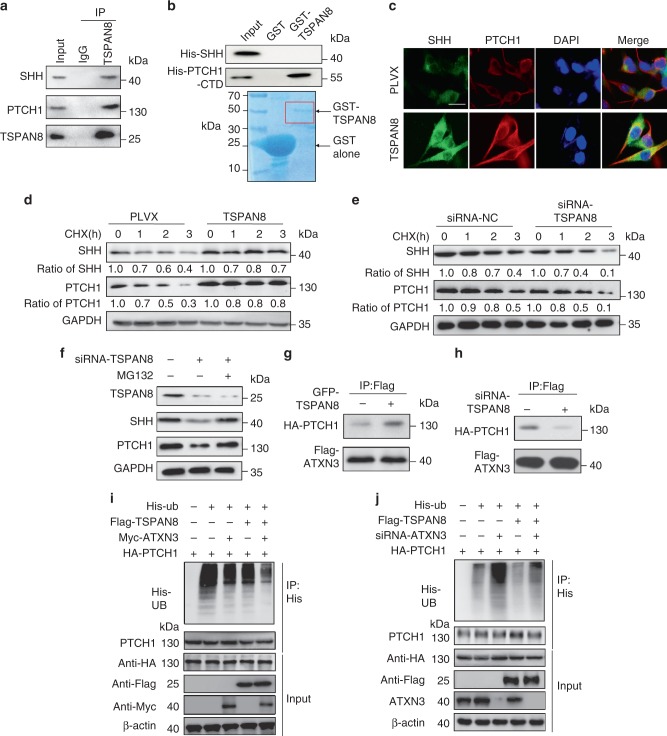
TSPAN8 stabilizes the expression of PTCH1 by recruiting ATXN3. **a** MCF7 cell lysates were immunoprecipitated with control rabbit IgG or anti-TSPAN8 and immunoblotted with anti-SHH or anti-PTCH1 antibodies. Ten percent of the cell extracts was loaded as an input. **b** GST pulldown assay was performed by mixing purified GST-TSPAN8 or GST with the lysates of HEK293T cell expressing His-SHH or His-PTCH1-CTD (C-terminal domain of PTCH1), then immunoblotting analyses were performed (top panel). Coomassie blue staining of GST-TSPAN8 and GST was performed (bottom panel). **c** Immunofluorescence analysis of MCF7 cells with or without *TSPAN8* overexpression was performed. DAPI was used to stain the nuclei. Scale bar = 30 μm. **d** MCF7 cells with or without *TSPAN8* overexpression were treated with CHX (100 μg/ml) for different periods of time. Quantitation was done by densitometry with Image J Software. SHH and PTCH1 band intensities were normalized according to the levels of GAPDH, SHH, and PTCH1 in the CHX-untreated cells (*n* = 3 per group, right). **e** MCF7 cells transfected with control siRNA or *TSPAN8* siRNA were treated with 100 μg/ml CHX for the indicated periods of time then immunoblotting analyses were performed with the indicated antibodies. **f** MCF7 cells transfected control siRNA or *TSPAN8* siRNA followed by treatment with or without MG132 (20 μM, 8 h). Immunoblotting analyses were performed with the indicated antibodies. **g** A vector expressing Flag-*ATXN3* was co-transfected with a vector expressing GFP or GFP-*TSPAN8* in HEK293T cells. Immunoprecipitation and immunoblotting analyses were performed with the indicated antibodies. **h** HEK293T cells were transfected with control siRNA or *TSPAN8* siRNA together with a Flag-*ATXN3* plasmid. Immunoprecipitation and immunoblotting analyses were performed with the indicated antibodies. **i**, **j** MCF7 cells expressing Myc-*ATXN3* (**i**) or *ATXN3* shRNA (**j**) were treated with MG132 (20 μM for 8 h) and SHH (100 ng/ml for 6 h). The whole-cell lysate was subjected to pulldown with His beads. All panels are representative results from three or more independent experiments

To determine the mechanism underlying TSPAN8-mediated activation of the Hh signaling pathway, we performed co-immunoprecipitation analyses and found that HA-TSPAN8 interacted with SHH (Supplementary Fig. 4a) and PTCH1 (Supplementary Fig. 4b). In addition, endogenous TSPAN8 was also associated with endogenous SHH and PTCH1 (Fig. 4a). The most divergent regions of PTCH1 are the central intracellular loop and the intracellular C-terminal domain (CTD), which is physically associated with the ubiquitin E3 ligases^27^. A GST pulldown assay demonstrated that the purified recombinant GST-TSPAN8 directly bound to purified His-PTCH1-CTD, but not to His-SHH (Fig. 4b). Of note, the overexpression of *TSPAN8* in 293T cells, which increased the expression levels of SHH and PTCH1 (Supplementary Fig. 4c), promoted the colocalization of SHH and PTCH1 (Fig. 4c).

To determine the mechanism underlying TSPAN8-upregulated SHH and PTCH1, we performed real-time PCR analyses and found that *TSPAN8* expression increased the mRNA levels of *SHH* and *PTCH1* (Fig. 3a, Supplementary Fig. 4d). However, treatment of MCF-7 cells with actinomycin D, which blocked *SHH* and *PTCH1* gene transcription (Supplementary Fig. 4d), did not abrogate TSPAN8-induced upregulation of SHH and PTCH1 protein, suggesting an involvement of protein stability regulation in this upregulation (Supplementary Fig. 4e). Consistent with this finding, treatment of MCF-7 cells with protein synthesis inhibitor cycloheximide (CHX) showed that *TSPAN8* overexpression stabilized PTCH1 and SHH expression by prolonging the half-lives of both proteins (Fig. 4d). In contrast, *TSPAN8* depletion decreased PTCH1 and SHH expression with shortened half-lives (Fig. 4e, Supplementary Fig. 4f). It is noteworthy, however, that this decrease was blocked by the proteasome inhibitor MG132 treatment (Fig. 4f). Overall, these results indicate that TSPAN8 enhances the stability of PTCH1 and SHH by preventing their proteasome-dependent degradation.

It is known that PTCH1 stability is regulated through ubiquitination-dependent proteasomal degradation^26^. We conducted mass spectrometry analyses of immunoprecipitated TSPAN8 and displayed that the ATXN3 deubiquitinase was an interacting protein. This association was further validated by co-immunoprecipitation analyses (Supplementary Fig. 4g–h). Intriguingly, the results of a GST pulldown assay with purified GST-ATXN3 revealed that ATXN3 interacted with both His-TSPAN8 and His-PTCH1 (Supplementary Fig. 4i). Importantly, *TSPAN8* overexpression enhanced the binding of ATXN3 to PTCH1 in MCF-7 cells, whereas *TSPAN8* depletion reduced their interaction (Fig. 4g–h). These results suggest that TSPAN8 facilitates the binding of ATXN3 to PTCH1 to reduce the degradation of PTCH1.

We next examined the regulation of PTCH1 ubiquitination by TSPAN8. Overexpression of *TSPAN8* or *ATXN3* decreased PTCH1 polyubiquitination, which was further reduced by co-expression of *TSPAN8* and *ATXN3* (Fig. 4i). In contrast, *ATXN3* depletion increased PTCH1 polyubiquitylation and alleviated the inhibitory effect of *TSPAN8* on PTCH1 polyubiquitylation (Fig. 4j). In addition, *ATXN3* depletion promoted the turn-over and degradation of PTCH1 whereas overexpression of *ATXN3* in endogenous *ATXN3*-depleted MCF7 cells prolonged half-life of PTCH1 and stabilized PTCH1 (Supplementary Fig. 4j–k). These results indicate that TSPAN8 stabilizes PTCH1 expression by promoting the binding of ATXN3 to PTCH1, leading to deubiquitylation of PTCH1.

### TSPAN8 promotes SHH-induced SMO phosphorylation

**Fig. 5:**
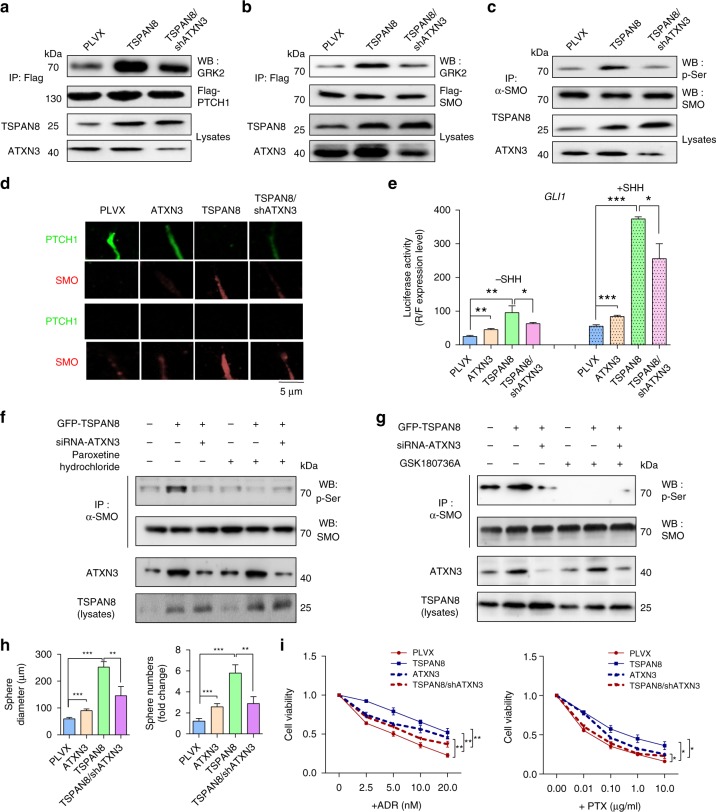
TSPAN8 promotes SHH-induced SMO phosphorylation. **a**, **b** Flag-PTCH1 (**a**) or Flag-SMO (**b**) was immunoprecipitated from MCF7 cells with or without *TSPAN8* overexpression in the presence or absence of *ATXN3* shRNA. Cells were treated with 100 ng/ml SHH for 6 h. Immunoblotting analyses were performed with the indicated antibodies. PLVX, lentiviral stable transfection plasmid pLVX-HA-IRES-Puro. **c** Endogenous SMO was immunoprecipitated from MCF7 cells with or without *TSPAN8* overexpression in the presence or absence of *ATXN3* shRNA. Cells were treated with 100 ng/ml SHH for 6 h. Immunoblotting analyses were performed with the indicated antibodies. P-Ser, phosphorylated serine. **d** MCF7 cells with or without overexpressing *ATXN3* or *TSPAN8* or MCF7 cells with overexpressing *TSPAN8* and *ATXN3* shRNA were left untreated or treated with 100 ng/ml SHH. Immunofluorescent studies were performed with the indicated antibodies. **e** MCF7 cells with or without overexpressing *ATXN3* or *TSPAN8* or MCF7 cells with overexpressing *TSPAN8* and *ATXN3* shRNA were transfected with a *GLI1* promoter–luciferase reporter plasmid. These cells were left untreated (left) or treated (right) with 100 ng/ml SHH. Luciferase activity was measured and normalized to Renilla luciferase activity (*n* = 3 per group). Two-tailed unpaired Student’s *t* test was performed. *P < 0.05, **P < 0.01, and ***P < 0.001. **f**, **g** MCF7 cells with or without expressing GFP-*TSPAN8* in the presence or absence of *ATXN3* siRNA expression were treated with 20 μM Paroxetine hydrochloride (**f**) or 2 μM GSK180736A (**g**) together with 100 ng/ml SHH for 6 h. Cellular extracts were immunoprecipitated with anti-SMO antibody, then immunoblotting analyses tested the serine-phosphorylation expression level. **h** Histograms show the mean numbers and diameters of spheres formed by MCF7 cells expressing the indicated proteins and *ATXN3* shRNA. **i** MCF7 cells expressing the indicated proteins and *ATXN3* shRNA were treated with ADR (left) or PTX (right) with the different concentrations for 24 h. The cell viabilities were determined. Cell numbers with no drug was used as control (*n* = 3 per group). Two-tailed unpaired Student’s *t* test was performed. *P < 0.05, **P < 0.01, and ***P < 0.001

Activated Sonic Hedgehog leads to SMO phosphorylation by PKA, CK1α, and GRK2, and subsequent ciliary localization and activation of GLIA^12^. We showed that *TSPAN8* overexpression enhanced the SHH-induced binding of GRK2 protein kinase to PTCH1 (Fig. 5a) and SMO (Fig. 5b), SMO phosphorylation (Fig. 5c), SMO accumulation in cilia (Fig. 5d), and the transcriptional activity of *GLI1* (Fig. 5e) in MCF-7 cells. Nevertheless, these increases were abrogated by *ATXN3* depletion. Of note, GRK2 inhibitor paroxetine hydrochloride (Fig. 5f) and GSK180736A (Fig. 5g) blocked the TSPAN8-induced SMO phosphorylation, suggesting that TSPAN8 enhances the binding of GRK2 to PTCH1 and SMO, leading to enhanced SMO phosphorylation. It is worth noting that *ATXN3* depletion reduced *TSPAN8* overexpression-enhanced MCF7 sphere formation efficiency (Fig. 5h) and resistance of these cells to chemotherapeutic agents (Fig. 5i). Similarly, depletion of *TSPAN8* or *ATXN3* in primary cancer cells derived from breast cancer patients decreased these cells sphere formation efficiency and enhanced their sensitivity to chemotherapeutic agents (Supplementary Fig. 5a, b). These results indicate that TSPAN8-enhanced and ATXN3-mediated PTCH1 expression promotes the binding of GRK2 to PTCH1 and SMO, leading to enhanced SMO phosphorylation, activated *GLI1* transcriptional activity, enhanced cancer cell stemness, and resistance to chemotherapeutic agents.

### TSPAN8 promotes tumorigenesis in mice

**Fig. 6:**
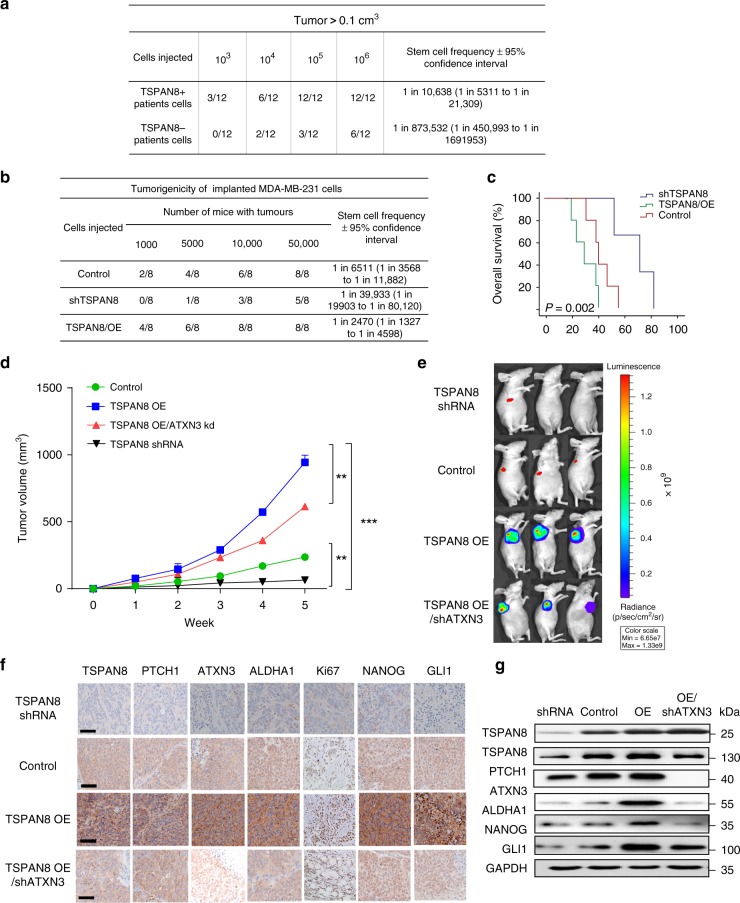
TSPAN8 promotes tumorigenesis in mice. **a** Flow cytometry was used to separate TS^+^ from TS^−^ tumor cells derived from three human breast cancer patients. These cells were subcutaneously injected (10^3^, 10^4^, 10^5^, 10^6^ cells per mouse) into NOD/SCID mice. Tumor formation ability and stem cell frequency were analyzed. **b**, **c** MDA-MB-231 cells with or without *TSPAN8* overexpression or depletion were subcutaneously injected (1000, 5000, 10,000, 50,000 cells per mouse) into 6-week-old female nude mice. Tumor formation ability and stem cell frequency were analyzed (**b**). Kaplan–Meier survival analysis was performed (**c**). *P* values were calculated by log-rank test (*n* = 15 mice, *P* < 0.001). **d**–**g** Nude mice were subcutaneously injected with 1 × 10^7^ Luc-MDA-MB-231 cells with or without depletion or overexpression of *TSPAN8* in the presence or absence of *ATXN3* shRNA expression. Tumor volumes were measured every week (**d**). Representative image of mice with xenograft tumors (**e**) and IHC staining of tumor samples (scale bar = 20 μm) (**f**) are shown. The expression levels of the indicated proteins in these tumors were examined by immunoblotting analyses (**g**). Each bar represents the mean ± SD. **P < 0.01, ***P < 0.001, 2-way ANOVA

To examine the role of TSPAN8 in tumorigenesis, we injected 1 × 10^3^–1 × 10^6^ of TS^+^ or TS^−^ primary breast cancer cells isolated from three patients’ breast cancer specimens into the breast fat pad of NOD/SCID mice to conduct a limiting dilution type assay. We found rapidly growing tumors mice injected with TS^+^ cells, and the tumor-forming frequency reached 100% when 10^5^ TS^+^ cells were injected (Fig. 6a). In contrast, injection of 10^5^ TS^−^ cells only had 25% tumor-forming frequency (Fig. 6a). The survival studies showed that TS^+^ cells injection shortened survival time of mice than TS^−^ cells injection (Supplementary Fig. 6a, b). In addition, based on the numbers of mice that had tumors > 0.1 cm^3^, we used the ELDA software^28^ to calculate stem cell frequencies. We found that the TS^+^ cells displayed a great increase in stem cell frequency (1:10,639) compared with TS^−^ cells (1:873,532) (Fig. 6a).

To further examine the role of TSPAN8- and ATXN3-regulated Hh signaling in tumor formation, we injected mice with MDA-MB-231 cells with or without depletion or overexpression of *TSPAN8* in the presence or absence of expression of *ATXN3* shRNA. *TSPAN8* overexpression markedly enhanced the tumor-forming ability and stem cell frequency (Fig. 6b) and shortened mouse survival time (Fig. 6c), whereas *TSPAN8* depletion reduced the tumor-forming ability, stem cell frequency and prolonged mouse survival time compared with control group. Our quantitative results showed that *TSPAN8* depletion reduced tumor growth. In contrast, *TSPAN8* overexpression-enhanced tumor growth, but this enhancement was inhibited by the expression of *ATXN3* shRNA (Fig. 6d, e). Immunohistochemistry (IHC) analyses showed that the TSPAN8 expression levels were correlated with the levels of Ki67, PTCH1, GLI1, ALDHA1, and NANOG, and the TSPAN8-enhanced expression of these proteins was reduced by *ATXN3* shRNA expression (Fig. 6f). These findings were further validated by immunoblotting analyses of these tumor specimens (Fig. 6g). Similar inhibitory effect of *TSPAN8* depletion on growth of mouse tumors derived from primary cancer cells of breast cancer patients was also observed (Supplementary Fig. 6c-e). These findings strongly confirm TSPAN8 promotes the activation of Sonic Hedgehog signaling and tumorigenesis by ATXN3-mediated stabilization of PTCH1 in vivo.

We next used a genetic approach with a therapeutic potential to modulate TSPAN8 expression in tumor. We intratumorally injected lentivirus expressing *TSPAN8*-shRNA, which inhibited MDA-MB-231 cell-derived tumor growth in mice (Supplementary Fig. 6f). The infection efficiency reached about 90%, which was determined by lentivirus-expressed GFP in tumor tissues (Supplementary Fig. 6g). IHC and immunoblotting analyses showed corresponding reduction of TSPAN8 in tumor tissues expressing *TSPAN8*-shRNA (Supplementary Fig. 6h). In addition, injection of lentivirus expressing *TSPAN8*-shRNA significantly reduced the PyMT-MMTV-induced breast tumor growth in mice (Supplementary Fig. 6i, j). These results indicate that TSPAN8 can be a molecular target for treating breast cancer.

### TSPAN8 correlates with PTCH1, SHH, and ATXN3 expression

**Fig. 7:**
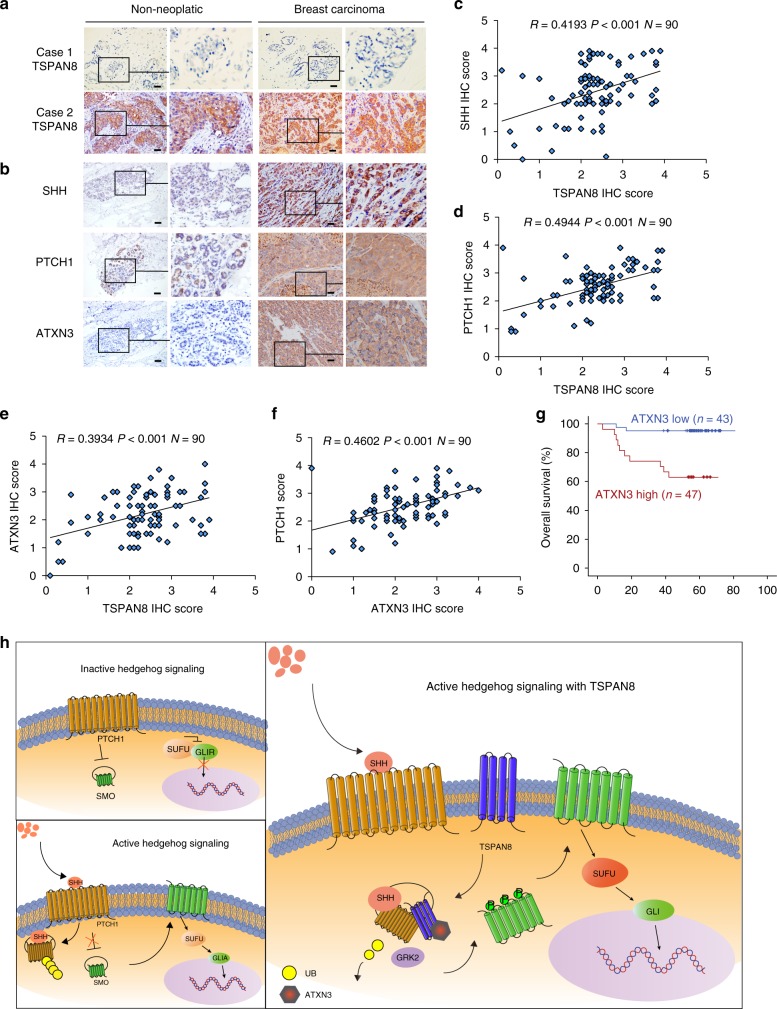
TSPAN8 correlates with PTCH1, SHH, and ATXN3 expression. **a** Representative IHC images of TSPAN8 in breast tumors (*n* = 20) and their matched adjacent tissues. **b** Representative IHC images of the expression levels of SHH, PTCH1, and ATXN3 in breast tumors. **c**–**f** Positive correlation of TSPAN8 expression with SHH (**c**), PTCH1 (**d**), and ATXN3 (**e**) expression and positive correlation between PTCH1 and ATXN3 expression (**f**) are shown. *P < 0.05, **P < 0.01, ***P < 0.001. The *P* values and correlation coefficient were analyzed as indicated. **g** Kaplan–Meier plot of survival from 90 breast cancer patients is shown. A log-rank test was used to calculate the difference between the two groups. **h** A mechanism of TSPAN8-enhanced Sonic Hedgehog signaling pathway. The interaction between TSPAN8 and PTCH1 leads to recruitment of ATXN3 deubiquitinating enzyme to the SHH-PTCH1 complex and subsequent deubiquitylation of PTCH1 and inhibition of proteasome-mediated degradation of SHH and PTCH1. Stabilized SHH and PTCH1 promote the binding of protein kinase GRK2 to SMO, phosphorylation and translocation of SMO to cilia, GLI1 activation for downstream gene expression, cancer cell stemness, as well as tumor formation in mice

To determine the clinical relevance of TSPAN8- and ATXN3-regulated PTCH1/SHH stability, we performed IHC staining of 90 breast cancer specimens and found that the expression of TSPAN8 in the tumor tissue was higher than that in the adjacent non-tumor tissue (Fig. 7a). In addition, a positive association between the expression levels of TSPAN8 and SHH, as well as between PTCH1 and ATXN3, in breast cancer specimens was observed (Fig. 7b). Pearson correlation analysis results demonstrated that these correlations were significant (Fig. 7c, d, and e) and that ATXN3 expression was positively correlated with PTCH1 expression (Fig. 7f). In addition, the high levels of ATXN3 expression were correlated with poor prognosis in breast cancer patients (Fig. 7g). These experiments strongly suggest that TSPAN8 together with ATXN3 promote the Hedgehog signaling and breast cancer progression by enhancing PTCH1 and SHH expression.

## Discussion

TSPAN8 has been linked to tumor growth and metastasis^16,17,19,20^. However, the exact function of TSPAN8 on the regulation of critical cellular activity and cancer stemness remains unclear. We demonstrated here that TSPAN8 overexpression promoted the expression of stem cell markers, like ALDHA1, increased the CD44^+^/CD24^−^ cell ratio, and enhanced the expression of pluripotency transcription factors including SOX2, OCT4, and NANOG. Of note, TSPAN8 expression was high in breast CSCs derived from patients who were resistant to conventional therapies and was further increased upon treatment with chemotherapeutic reagents. Analyses of gene expression profiles in our study revealed that *PTCH1*, *SHH*, and *GLI1*, which are the Hh pathway-targeted genes, were upregulated by TSPAN8 expression. In our attempts to determine the mechanism underlying this regulation, we found that TSPAN8 directly interacted with PTCH1 and subsequently recruited ATXN3 to the SHH-PTCH1 complex. ATXN3 deubiquitylated PTCH1 and inhibited the proteasome-mediated degradation of the SHH-PTCH1 complex. Moreover, stabilized SHH and PTCH1 promoted the binding of GRK2 protein kinase to SMO. This interaction resulted in GRK2-enahnced SMO phosphorylation, the release of SMO from the binding of PTCH1, and its translocation of SMO to cilia, leading to the activation of GLI1 and subsequent induction of downstream genes expression. Consequently, the TSPAN8 expression promoted cancer cell stemness, resistance of CSCs to chemotherapeutic agents, and tumor formation in mice (Fig. 7h).

In the absence of corresponding ligands, PTCH1 binds to SMO and suppresses its activation. In the presence of its ligands, PTCH1’s inhibitory effect was alleviated by protein kinases-mediated SMO phosphorylation, causing the movement of SMO from the plasma membrane near the primary cilium along the membrane into the ciliary membrane through a lateral transport pathway^29^. Our results showed that TSPAN8 enhanced the expression levels of PTCH1 and SHH together with increased SHH-bound PTCH1, thereby promoting the recruitment of GRK2, a protein kinase, to the complex to phosphorylate SMO. Importantly, this regulation is mediated by deubiquitylation of PTCH1 by ATXN3.

Our reports revealed that TSPAN8 expression enhances Hh signaling and contributes to cancer cell stemness. As demonstrated by previous publications, elevated Hh signaling and cancer stem cell play instrumental roles in drug resistance^30^. Thus, our results suggested that NAC-increased TSPAN8 expression enable cancer cells resistant to treatment through enhanced Hh signaling and cancer cell stemness. Given that CD44^+^/CD24^−^ are also present in TSPAN8^−^ cells albeit at a low level, TSPAN8 alone may not be sufficient to demarcate a stem cell population. Considering the complexity and heterogeneity of CSCs, TSPAN8 can be used as a molecular marker in combination with other CSCs markers, such as ALDH1 and CD44^+^/CD24^−^, to define cancer cell stemness.

PTCH1 overexpression has been identified in a wide variety of cancers, including breast tumor, pancreatic neuroendocrine tumors, lung cancer, and gastric cancer^31–35^. Besides, the expression levels of *SHH*, *PTCH1*, and *GLI3* mRNA were significantly increased in gastric CSCs compared with non-CSCs^36^. Our analyses of human breast cancer specimens revealed positive correlation among the expression levels of TSPAN8, PTCH1, SHH, and ATXN3. Importantly, TSPAN8 and ATXN3 expression levels were associated with poor prognosis in breast cancer patients. Thus, our findings unearth an instrumental mechanism underlying the activation of the Sonic Hedgehog signaling. The establishment of the critical role of TSPAN8 in promoting cancer cell stemness, breast cancer development, and resistance to chemotherapy, makes TSPAN8 a molecular target for therapy of human malignancies.

## Methods

### Human samples and cancer stem cells

The experiment with human tissues was authorized by the Human Ethics Committee of Shanghai General Hospital, Shanghai Jiao Tong University School of Medicine (Shanghai, China). All subjects provided written informed consent. A piece of approximate 1 cm^3^ breast cancer tissue was removed during an operation and washed with DMEM/F12 (1:1) to remove the adipose tissue. Next, the tissue was cut into 1 mm^3^ pieces, followed by the addition of 100 U/mL III collagenase, 100 U/mL penicillin, 150 U/mL hyaluronidase and DMEM/F12 (1:1) medium and incubation for 12–18 h at 37 °C. Trypsin was added to the pellet. After digestion for 10 min, the pellet was gently and repeatedly swirled, filtered through a sieve, and centrifuged. The resulting pellet was added to a stem-cell culture medium, which was composed of DMEM/F12, supplemented with 20 ng/mL recombinant human EGF (rhEGF) and 20 ng/mL of basic fibroblast growth factor (bFGF) together with B27 and ITS (Insulin-Transferrin-Selenium). Replace half of the medium with fresh medium every 2 days.

### Cell lines, culture conditions, and treatments

Human breast cancer cell lines MDA-MB-231, HCC-1954, MCF-7, T47D, BT474, and MDA-MB-468 were obtained from ATCC and grown in RPMI 1640 medium (HyClone) supplemented with 1% penicillin-streptomycin, 10% fetal bovine serum at 37 °C with 5% CO_2_. The sphere (cancer stem cell) medium was similar to stem-cell culture medium of human samples. All cell lines were checked by STR profiling and mycoplasma was tested every 2 weeks.

### Plasmids and lentivirus production

Transient transfection of siRNA was performed with Lipofectamine RNAiMAX (Invitrogen). Supplementary Table 1 lists the siRNA sequences used in the article. The instantaneous transfection of plasmid DNA was performed by using a DNA transfection reagent (Lipofectamine lipo3000; Invitrogen). Cells were collected 48 h after transfection. If the protein needed to be quantified, 0.7 M beta-mercaptoethanol was added to the protein sample after quantification, and then boiled for 10 min at 95 °C. After transfection, microRNeasy kit (Qiagen) was used to extract cellular RNA or to lyse cells in SDS buffer. After RNA reverse transcription, the appropriate sequence was amplified by using the cDNA library.

For the lentivirus packaging, *TSPAN8* and *ATXN3* were inserted into the viral skeleton plasmid EGFP-tagged 3 × Flag-PGK-Puro. Annealing and connection of the shRNAs were performed before insertion into the viral skeleton plasmid pLKO.1-Puro. The constructed vectors were subsequently transformed into HEK293T cells using lentivirus packaging reagents (ADDGENE, Shanghai, China) according to the manufacturer’s instructions. The shRNA sequences in our research are shown in Supplementary Table 3.

### Immunoprecipitation and immunoblotting analyses

Co-IP experiments were performed for protein A/G, anti-HA, or anti-Flag magnetic beads (IP) (Biotool, USA) according to the instructions. The harvested cells were lysed in RIPA buffer (Beyotime) at 4 °C for IP and western blotting. Cell lysates were incubated with magnetic beads to prepare bead-antibody complexes (10 μg antibody and 50 μL protein A/G magnetic beads) for 12 h at 4 °C. After washing with the elution buffer, the protein complexes were boiled and subjected to western blotting.

Protein samples were separated by SDS-PAGE, transferred to a polyvinylidene difluoride membrane (PVDF), blocked and tested with the indicated primary antibody and horseradish peroxidase (HRP)-conjugated secondary antibody (Santa Cruz Biotechnology, Santa Cruz, CA, USA). Chemiluminescent HRP substrates (Millipore, Billerica, MA, USA) were used to visualize antibody binding. The protein levels of Flag-TSPAN8 in different samples immunoprecipitated by anti-Flag magnetic beads were detected by immunoblotting analysis using an antibody against Flag, followed by quantification using the image analysis software Imagine J (NIH, MD, USA). All the bands that are not cropped are in Supplementary Figs. 7–22.

The following antibodies were used: TSPAN8 (1:1000, Abcam, ab70007), ALDHA1 (1:1000, Proteintech, 15910-1-AP), SHH (1:1000, Proteintech, 20697-1-AP), IHH (1:1000, Proteintech, 13388-1-AP), Phosphoserine (1:100, Abcam, ab15556), PTCH1 (1:1000, Proteintech, 17520-1-AP), GLI1 (1:1000, Proteintech, 66905-1-lg), SMO (1:1000, Proteintech, 20787-1-AP), NANOG (1:1000, Proteintech, 14295-1-AP), SOX2 (1:1000, Proteintech, 1-1064-1-AP), and ATXN3 (1:1000, Proteintech, 13505-1-AP).

### Confocal immunofluorescence microscopy

Cells harvested on coverslips were washed with phosphate buffered saline (PBS) before fixation for 15 min in 4% formalin. The cells were then treated with 0.25% Triton X-100 for 15 min, followed by washes with PBS. Next, the cells were blocked with 1% bovine serum albumin (BSA) for 1 h, followed by incubation with primary antibodies at room temperature for 2 h. After PBS washes for three times, the cells were incubated with an anti-mouse IgG conjugated with Alexa Fluor® 568 (1:1000) (Thermo scientific) and an anti-rabbit IgG conjugated with Alexa Fluor® 488 (1:1000) (Thermo scientific) for 1 h. The resulting signals were visualized using a confocal laser-scanning microscope (Olympus BX61, Tokyo, Japan).

### Gene expression analysis

Cellular total RNA was isolated with an E.Z.N.A total RNA Kit I (Omega Bio-Tek, Inc., Norcross, GA, USA). The cDNA was synthesized with a PrimerScript RT reagent Kit (Takara, Ostu, Shiga, Japan). The real-time PCR reaction was performed according to the protocol of the SYBR Premix Ex Taq kit (Takara) and using a StepOnePlus Real-Time PCR System (Applied Biosystems, USA). The fluorescence data of the detected genes were normalized to the expression of cyclophilin B (CB) using the 2-ΔΔCT method. The primers utilized in our investigation were listed in Supplementary Table 4.

### Luciferase reporter assay

The human GLI1 gene promoter was fused to the luciferase gene into the pGL3 vector (Promega, WI, USA). Cells were cultured in 24-well culture dishes (1.5 × 10^4^/well) and transfected with the luciferase reporter vector together with a renilla luciferase plasmid at a ratio of 10:1. The luciferase activity of the cells was analyzed with a dual-luciferase assay system (Promega). The relative levels of luciferase activity were normalized to the renilla luciferase activity levels.

### Cell growth assay

Cells were seeded on 24-well plates in 1% FBS DMEM (5000 cells per well, three parallel wells). Then, the cells were harvested after the transfection at different time points, and their number on each plate was counted. CCK-8 was employed to identify dead cells when counting.

### Colony formation assay

Cells were suspended in 0.3% agarose (Sigma-Aldrich) and placed at a density of 50 cells per well. After 3 weeks of cultivation, the cells were fixed and stained with 10% formalin and 0.1% crystal violet. The relative numbers of colonies were counted.

### Immunohistochemistry and tissue array

The human cancer tissue specimens were collected by surgical resection after obtaining consent. Tumor (5 × 3 × 3 mm) and normal adjacent tissues with a distance of 2 cm from the tumor (3 × 3 × 5 mm) were prepared. As shown in Supplementary Table 2, the detailed clinicopathological data were scored on the basis of the tumor classification of the American Joint Committee on Cancer (AJCC)/International Union Against Cancer (UICC) tumor staging system. The pathological types of paraffin-embedded slides were checked again by HE staining before immunostaining. Subcutaneous tumor tissues of nude mice were fixed in 4% paraformaldehyde, dehydrated, paraffin-embedded, and cut into 4 µm sections. Next, after transfection of 48 h, cells were fixed in 4% paraformaldehyde. The activity of endogenous peroxidase in the tissue sections or fixed cells was blocked with 3% hydrogen peroxide solution (Sangon Biotech). The antigens were retrieved, and the nonspecific binding was blocked by 4% normal goat serum (Gibco). Subsequently, tissue sections or cell coverslips were incubated with different primary antibodies, followed by incubation with HRP-conjugated goat anti-rabbit IgG (1:1000, Cell Signaling, MA, USA). Then, 3,3′-diaminobenzidine (DAB) chromogen substrate solution was utilized to visualize the results.

For the tissue microarray construction, all cancer specimens were histopathologically reevaluated and the representative areas were marked. The following antibodies were used in the IHC experiment: anti-TSPAN8 rabbit polyclonal antibody (1:100, Abcam, ab70007), anti-ALDHA1 rabbit polyclonal antibody (1:200, Proteintech, 15910-1-AP), anti-GLI1 rabbit polyclonal antibody (1:200, Proteintech), anti-PTCH1 rabbit polyclonal antibody (1:100, Proteintech, 17520-1-AP), anti-SHH rabbit polyclonal antibody (1:50, Proteintech, 20697-1-AP), anti-SOX2 rabbit polyclonal antibody (1:100, Proteintech, 11064-1-AP), and anti-Ki67 rabbit polyclonal antibody (1:200, Abcam, ab15580). The TSPAN8 expression in tissues was scored blindly and independently by two scientists. The scores for staining frequency (0 = 0%, 1 = 1%, 2 = 2–10%, 3 = 11–30%, 4 = 31–70%, and 5 = 71–100%) and intensity (0 = negative, 1 = weak, 2 = moderate, and 3 = strong staining) were added to obtain an overall staining score (OSS). An OSS of 0–2 was deemed low, 3–5 moderate, and 6–8 high. The average score for each slide was obtained by two independent pathologists, who calculated QS by multiplying the intensity score by the percentage of the staining area.

### FACS

Antibodies for the human antigens CD45 (Alexa-450, eBioscience), EpCAM (PE, eBioscience), CK (FITC, eBioscience), CD24 (PE, eBioscience), CD44 (APC, eBioscience), and TSPAN8 (FITC, eBioscience) were used for fluorescence-activated cell sorting (FACS). Antibodies are validated according to the manufacturer’s website. Briefly, tissues were mechanically chopped and were then digested at 37 °C for 45 min with collagenase-1. The resulting suspension was treated with DNAse at room temperature for 5 min, washed, and dissociated with trypsin/EDTA for 10 min at 37 °C, and filtered through a 70-μm filter. We used antibodies of EpCAM, CD45, and CK to seperate EpCAM^+^/CD45^−^/CK^+^ epithelial tumor cells from stromal cells (EpCAM negative) and blood cells (CD45 positive). Then, we used TSPAN8 antibodies to sort TSPAN8-negative and TSPAN8-positive cells. In order to detect the proportion of cancer stem cells, CD44^+^/CD24^−^ flow cytometry analysis and FACS was performed using dual-staining for CD24 and CD44 with propidium iodide exclusion of non-viable cells. Trypsin-digested cells were washed and centrifuged for 5 min at 800 × *g*, 1 µL of antibody was added to the samples, which were next shielded from light and left undisturbed for 15 min at 4 °C. Flow sorting was performed with a BD FACSAria II cell sorter (Becton Dickinson). The side scatter width versus side scatter region (SSC-W versus SSC-A) and the forward scatter width and forward scatter height (FSC-W versus FSC-H) were used to eliminate dead cells and cell clumps.

### GST pulldown assay

GST-fused TSPAN8 and ATXN3 were expressed in BL21 *E. coli* and purified using glutathione-sepharose 4B beads (GE Healthcare, Uppsala, Sweden) according to the standard protocols. Coomassie blue staining was performed to quantify its expression by a small amount of proteins. Resinous Ni-affinity (GE Healthcare) was used to purify His-tagged proteins. His-PTCH-CTD, His-SHH, or cell lysates were then mixed with purified GST-TSPAN8 or GST beads for 1 h at 4 °C. Then washed the beads 10 times with IP buffer before immunoblotting analyses.

### Xenograft tumor studies

All animal studies were conducted according to the guidelines provided by the Animal Ethics Committee of Shanghai Renji Hospital, Shanghai Jiao Tong University School of Medicine (Shanghai, China). MDA-MB-231 cells were subcutaneously inoculated of 5-week-old female nude mice from Shanghai Laboratory Animal Center. Tumors were measured and monitored every 4 days, the results of which were presented as mean ± SEM. The tumor tissues were collected, weighed, and photographed at the end of the experiments.

For the genetic therapeutic approach, 15 tumor-bearing nude mice were randomly divided into three groups, five in each group for intratumoral injection. The injection was performed by intratumoral multiple-point injection every 3 days. Treatment group: injection of TSPAN8-shRNA lentivirus 0.1 ml/(5 × 10^8^ TU/ml); negative control group: injection of NC-shRNA lentivirus 0.1 ml/(5 × 10^8^ TU/ml); blank control group: PBS 0.1 ml. The treatment was performed when the tumor grew to a diameter of 4–5 mm. PyMT-MMTV breast cancer spontaneous tumor-forming mice are from the Jackson Laboratory. Most PyMT-MMTV mice began to develop palpable tumors in the ninth week. Ten PyMT-MMTV mice were randomly divided into two groups. We performed intratumoral multiple-point injection with lentivirus as the method described above when the tumor grew to 4–5 mm in diameter. Treatment group: injection of TSPAN8-shRNA lentivirus 0.1 ml/(5 × 10^8^ TU/ml); negative control group: injection of NC-shRNA lentivirus 0.1 ml/(5 × 10^8^ TU/ml).

### Statistical analysis

All data were statistically analyzed with GraphPad Prism 6.0 and SPSS 20.0 software. Two-tailed *t* test was utilized to analyze the difference between the two groups. Pearson’s test was applied to determine the correlation between clinicopathological parameters and protein expression. Data were presented as mean ± SD or SEM. Differences at *P* < 0.05 were considered statistically significant.

### Reporting summary

Further information on research design is available in the Nature Research Reporting Summary linked to this article.

## Supplementary information

Supplementary Information

Reporting Summary

Source Data

## Data Availability

The data that support the findings of this study are available from the corresponding author upon reasonable request. Source data are provided as a Source Data file.
