# Supplementary material for: TSPAN8 promotes cancer cell stemness via activation of sonic Hedgehog signaling
Source: Nat Commun. 2019 Jun 28;10:2863. doi: 10.1038/s41467-019-10739-3 (PMC6599078; doi:10.1038/s41467-019-10739-3)
Supplement: Supplementary file 1 — Supplementary Information [file 41467_2019_10739_MOESM1_ESM.pdf]

Supplementary Information:

TSPAN8 Promotes Cancer Cell Stemness *via* Activation of Sonic Hedgehog Signaling

Zhu et al.

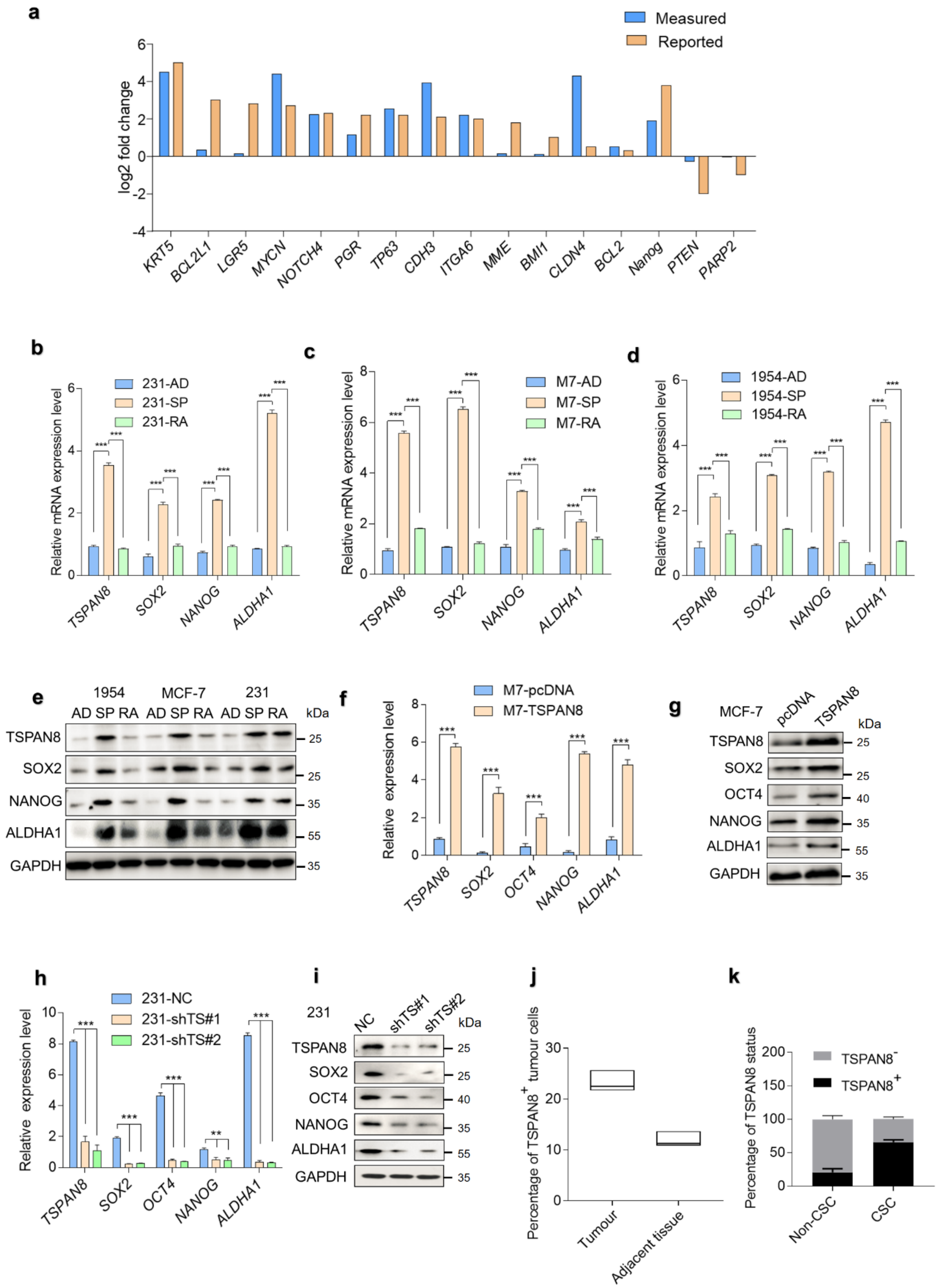
a
Measured
Reported
log2 fold change
6
4
2
0
-2
-4
KRT5
BCL2L1
LGR5
MYCN
NOTCH4
PGR
TP63
CDH3
ITGA6
MME
BMI1
CLDN4
BCL2
Nanog
PTEN
PARP2
b
231-AD
231-SP
231-RA
Relative mRNA expression level
TSPAN8
SOX2
NANOG
ALDHA1
c
M7-AD
M7-SP
M7-RA
d
1954-AD
1954-SP
1954-RA
e
1954
MCF-7
231
AD SP RA AD SP RA AD SP RA
kDa
TSPAN8
SOX2
NANOG
ALDHA1
GAPDH
f
M7-pcDNA
M7-TSPAN8
Relative expression level
OCT4
g
MCF-7
pcDNA
TSPAN8
h
231-NC
231-shTS#1
231-shTS#2
i
231
NC
shTS#1
shTS#2
j
Percentage of TSPAN8+ tumour cells
Tumour
Adjacent tissue
k
TSPAN8-
TSPAN8+
Percentage of TSPAN8 status
Non-CSC
CSC

Supplementary Figure 1. TSPAN8 regulates stemness-related makers.

(a) The data of the reported group were fold changes of low-burden metastatic tumor cells (basal/stem-like cells) relative to primary tumor cells. The data of the measured group were derived from the same batch of samples used in Figure 1a (only a portion of the results were shown because of image size limitation).

(b-d) qRT-PCR analyses of CSCs-associated genes in AD (adherent cells), SP (spheres) and RA (re-adherent cells). Spheres derived from adherent cells and re-adherent cells derived from spheres were cultured for a week. MDA-MB-231 (231) (b), MCF7 (c), HCC-1954 (1954) (d) breast cancer cells were used. Data are normalized to *GAPDH* expression and are presented as fold changes in gene expression relative to adherent cells. N = 3 biologically independent samples per group. $^{**}P < 0.01$, $^{***}P < 0.001$ by Student's t test. Bar graphs are shown as mean ± SD.

(e) Expression of TSPAN8, SOX2, NANOG and ALDHA1 in adherent cells, spheres, and re-adherent MDA-MB-231, MCF7 and HCC-1954 cells were examined with immunoblotting analyses. GAPDH was used as a loading control.

(f, g) qRT-PCR (f) and immunoblotting analyses (g) of TSPAN8, SOX2, OCT4, NANOG and ALDHA1 expression in MCF7 with or without *TSPAN8* overexpression were performed. GAPDH was used as loading control. N = 3 biologically independent samples per group. $^{***}P < 0.001$ by Student's t test. Bar graphs are shown as mean ± SD.

(h, i) qRT-PCR (h) and immunoblotting analyses (i) of TSPAN8, SOX2, OCT4, NANOG and ALDHA1 expression in MDA-MB-231 cells with or without *TSPAN8* depletion (two

different clones) were performed. GAPDH was used as loading control. N = 3 biologically independent samples per group. *$P < 0.05$, **$P < 0.01$, ***$P < 0.001$.

(j) Flow cytometry analyses of the ratios of TSPAN8^{+} cells derived from tumor and adjacent tissues of 10 human breast cancer patients were performed.

(k) Flow cytometry analyses was used to sort CD44^{+}/CD24^{-} cells and CD44^{-}/CD24^{+} cells from 10 human breast cancer patient tumor specimens. The ratios of TS^{+} cells in these cells were determined.

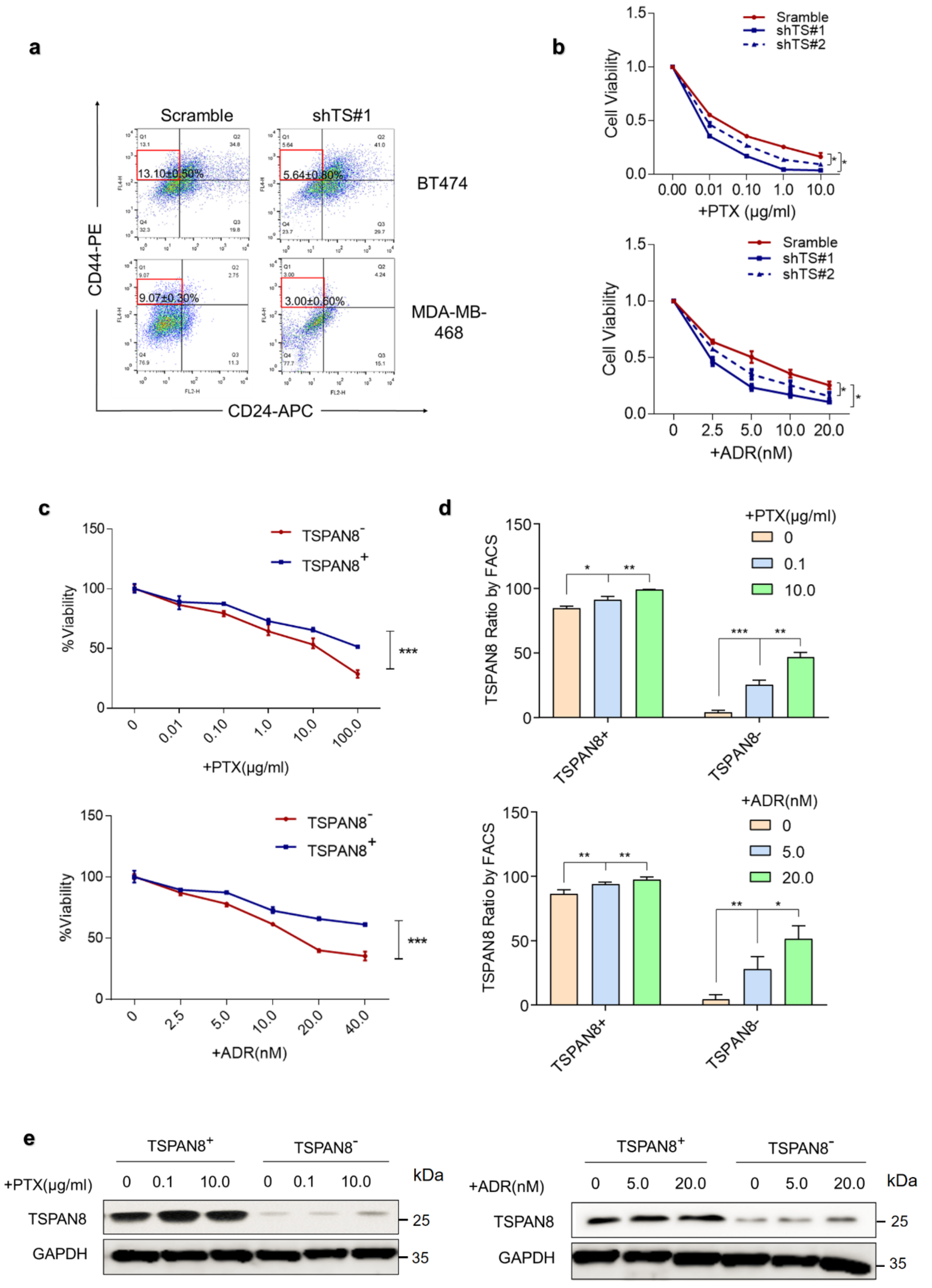
a
Scramble
shTS#1
13.10±0.50%
5.64±0.80%
BT474
9.07±0.30%
3.00±0.60%
MDA-MB-468
CD44-PE
CD24-APC
b
Cell Viability
Sramble
shTS#1
shTS#2
+PTX (μg/ml)
+ADR(nM)
c
%Viability
TSPAN8⁻
TSPAN8⁺
+PTX(μg/ml)
+ADR(nM)
***
d
TSPAN8 Ratio by FACS
+PTX(μg/ml)
0
0.1
10.0
+ADR(nM)
0
5.0
20.0
TSPAN8+
TSPAN8-
e
TSPAN8⁺
TSPAN8⁻
+PTX(μg/ml)
+ADR(nM)
0
0.1
10.0
5.0
20.0
kDa
TSPAN8
GAPDH
25
35

Supplementary Figure 2. Knockdown of *TSPAN8* decreases cancer cell stemness and drug resistance.

(a) Flow cytometry analysis of the ratios of $CD44^{+}/CD24^{-}$ in BT474 and MDA-MB-468 cells with or without *TSPAN8* shRNA expression was performed. N = 3 biologically independent samples per group.

(b) Effects of PTX and ADR at the indicated concentrations for 24 hours on viability of MCF7 cells with or without *TSPAN8* shRNA expression were determined. N = 3 biologically independent samples per group. **P* < 0.05 by Student's t test. Bar graphs are shown as mean ± SD.

(c) Effects of ADR and PTX at the indicated concentrations for 24 hours on viability of TS^{+} cells and TS^{-} cells were determined. N = 3 biologically independent samples per group. TS^{+} and TS^{-} cells were isolated from human breast cancer specimens. ****P* < 0.001 by Student's t test. Bar graphs are shown as mean ± SD.

(d) TS^{+} and TS^{-} cells were isolated from human breast cancer specimens, and the ratios of TS^{+} cells were calculated by flow cytometry analysis after treatment with ADR or PTX at the indicated concentrations. **P* < 0.05, ***P* < 0.01, ****P* < 0.001.

(e) TS^{+} and TS^{-} cells were isolated from human breast cancer specimens and treated with ADR or PTX at the indicated concentrations. Immunoblotting analyses were performed with the indicated antibodies.

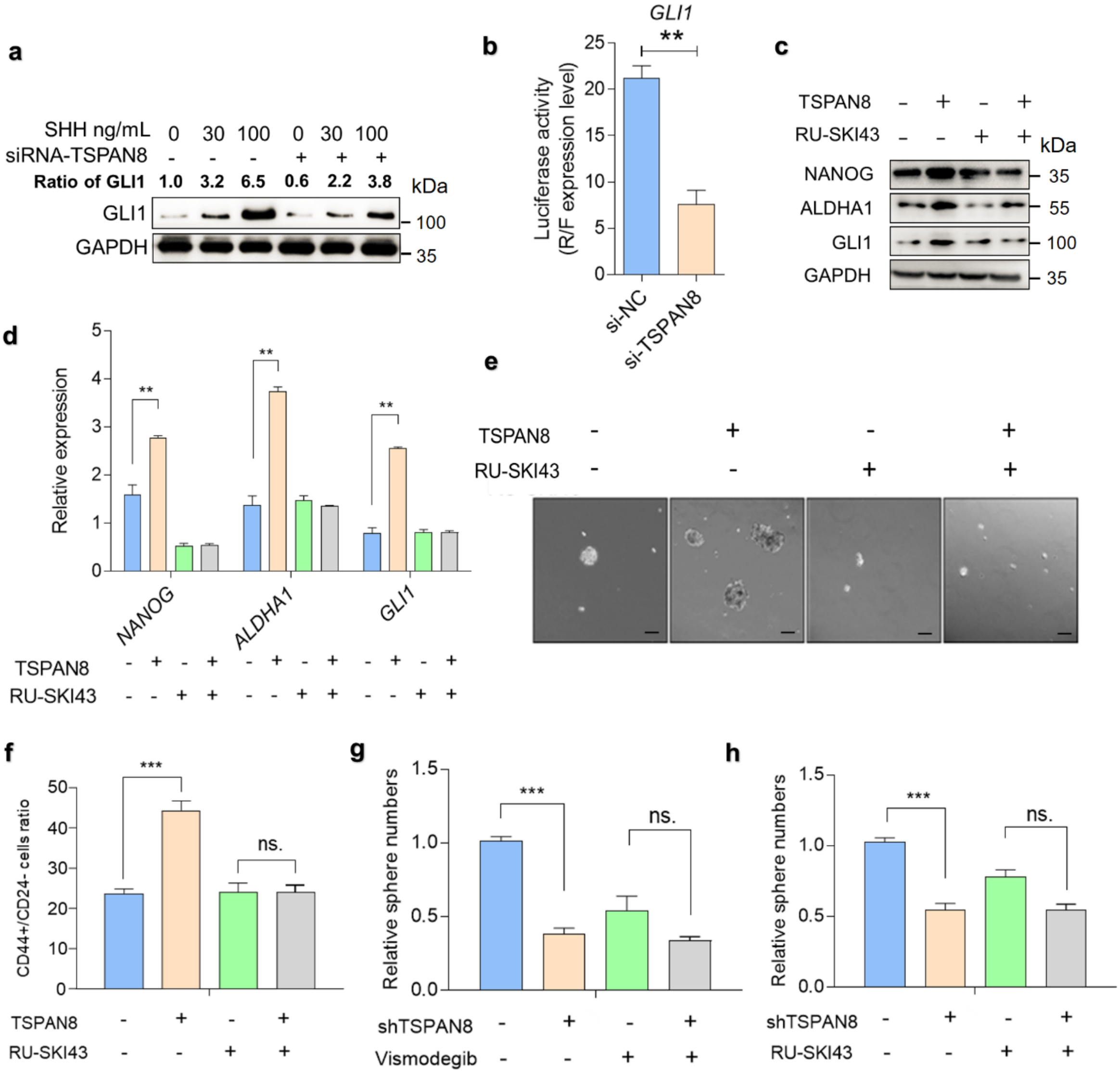

Supplementary Figure 3. Hh signaling pathway inhibitors abrogate TSPAN8 functions.

(a) MCF7 cell with or without *TSPAN8* shRNA expression were treated with SHH at the indicated dosages for 6 hours. Immunoblotting analyses were performed with the indicated antibodies.

(b) MCF7 cells with or without expressing *TSPAN8* shRNA were transfected with a *GLI1* promoter-luciferase reporter plasmid. Luciferase activity was measured and normalized to renilla luciferase activity. Empty vector was used as a control (N = 3 per group). Two-tailed unpaired Student's T-test was performed. $^{**}P < 0.01$.

(c) T47D cells with or without overexpressing *TSPAN8* were treated with or without RU-SKI43 for 6 hours. Immunoblotting analyses were performed with the indicated antibodies.

(d) T47D cells with or without *TSPAN8* overexpression were treated with or without RU-SKI43 for 6 hours. qRT-PCR analyses of the indicated gene expression were performed. Results between the two independent groups were determined by Student's t-test, and comparisons among three or more groups were determined by one-way analysis of variance (ANOVA) followed by Bonferroni's post hoc testing.

(e) T47D cells with or without *TSPAN8* overexpression were treated with or without RU-SKI43 for a week. Spheres were cultured for a week before counting. Scale bar = 50 μm.

(f) T47D cells with or without *TSPAN8* overexpression were treated with or without RU-SKI43 for 6 hours. Flow cytometry analysis of the ratios of $CD44^{+}/CD24^{-}$ in these cells was performed.

(g) MCF7 cells with or without *TSPAN8* depletion were treated with or without vismodegib for a week. Tumor sphere forming ability of the indicated cells with or without *TSPAN8* depletion was examined. Histograms show the mean numbers of spheres cultured. The results are presented as means ± SD from three independent experiments. Two-tailed unpaired Student's T-test was performed. ***$P < 0.001$.

(h) T47D cells with or without *TSPAN8* depletion were treated with or without RU-SKI43 for a week. Tumor sphere forming ability of the indicated cells with or without *TSPAN8* depletion was examined. The results are presented as means ± SD from three independent experiments. Two-tailed unpaired Student's T-test was performed. ***$P < 0.001$.

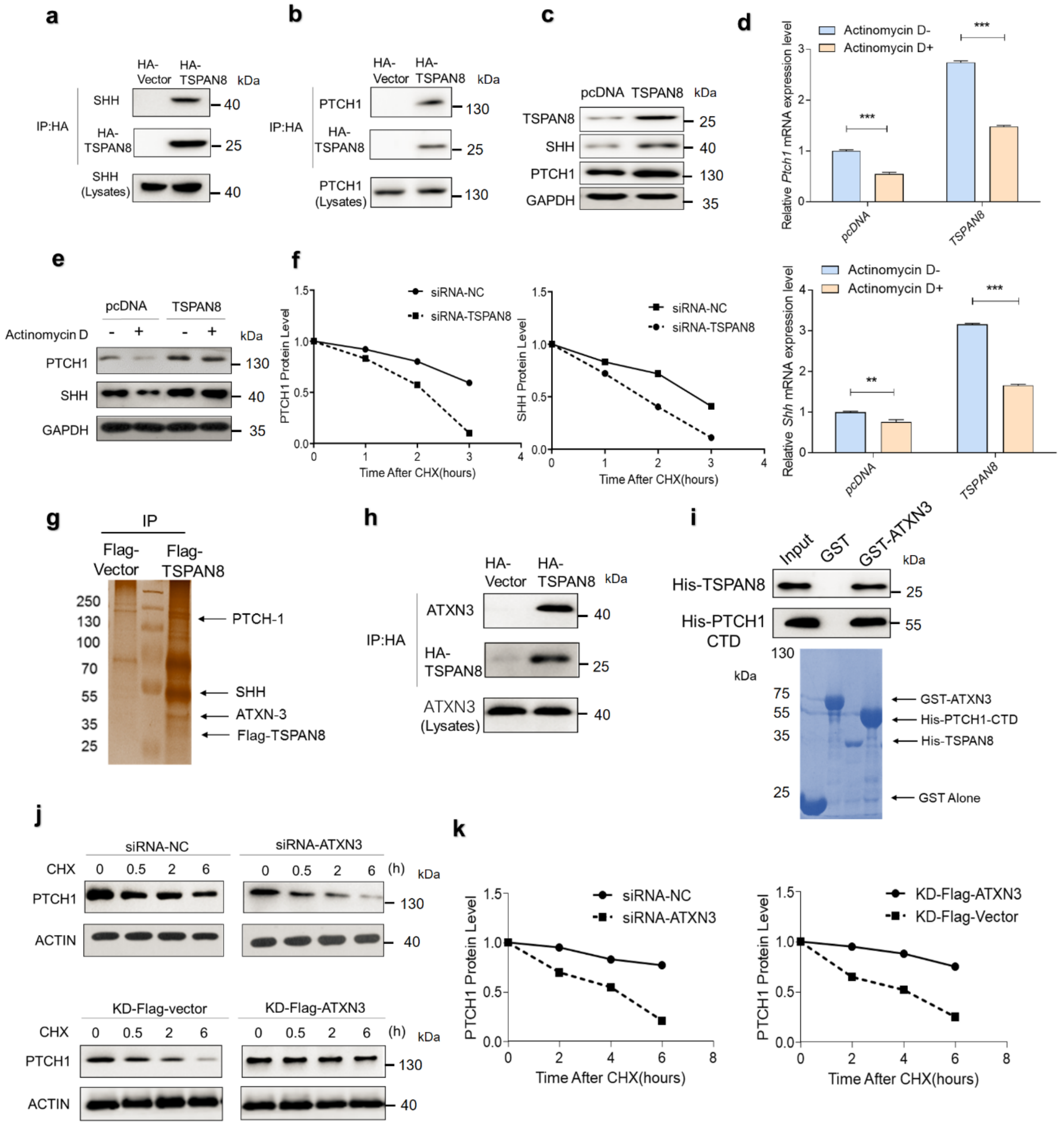

Supplementary Figure 4. ATXN3 is a deubiquitin enzyme of PTCH1.

(a, b) Lysates of HEK293T with or without expressing HA-TSPAN8 were immunoprecipitated with an anti-Flag antibody and immunoblotted with an anti-SHH (a) or anti-PTCH1 (b) antibody. Ten percent cell lysates as a control is shown.

(d-e) MCF7 cells with or without *TSPAN8* overexpression were treated without (c) or with 5 μg/ml actinomycin D for 6 h. qRT-PCR (d) and Immunoblotting (e) analyses of Hh signaling-related proteins were performed. Results between the two independent groups were determined by Student's t-test, and comparisons among three or more groups were determined by one-way analysis of variance (ANOVA) followed by Bonferroni's post hoc testing. *$P < 0.05$, **$P < 0.01$, ***$P < 0.001$.

(f) MCF7 cells with or without expressing *TSPAN8* siRNA were treated with 100 μg/ml cycloheximide. Quantitation was done by densitometry with Image J Software; PTCH1 and SHH band intensity was normalized to GAPDH, then normalized to the time = 0 controls.

(g) HEK293T cell lysates were incubated with purified Flag-TSPAN8 protein. Bound proteins were eluted and analyzed by SDS-PAGE. The silver staining shows the location of Flag-TSPAN8 and its associated protein PTCH1, SHH and ATXN3. Proteins identified in each group are indicated in the graph.

(h) Immunoprecipitation analyses of HEK293T cells transfected with HA-vector or HA-*TSPAN8* plasmids were performed. Lysates were immunoprecipitated with anti-HA beads and immunoblotted with an anti-ATXN3 antibody. Empty vector was used as a control.

(i) GST pull-down assays were performed by mixing purified GST-ATXN3 or GST with the lysates of HKT293T cell expressing His-TSPAN8 or His-PTCH1-CTD (C-terminal domain of PTCH1). Immunoblotting analyses and coomassie blue staining were performed.

(j, k) MCF7 cells with or without expressing *ATXN3* siRNA were treated with 100 μg/ml cycloheximide. Immunoblotting analyses with the indicated antibodies were performed (j). Quantitation was done by densitometry with Image J Software; PTCH1 band intensity was normalized to GAPDH, then normalized to the time = 0 controls (k).

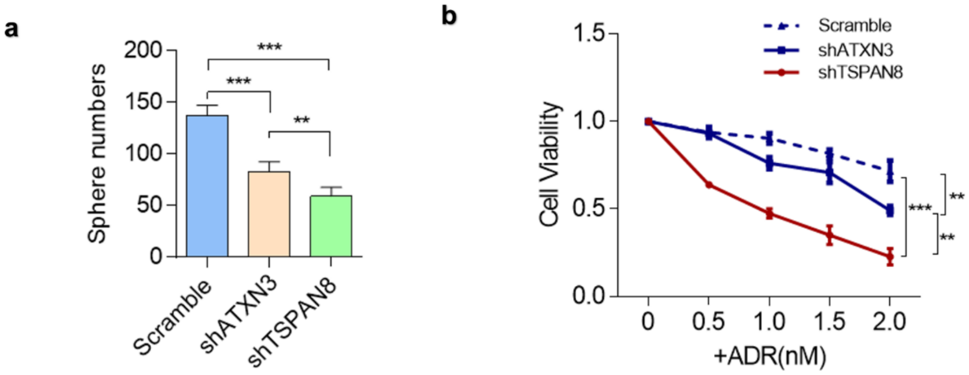

Supplementary Figure 5. TSPAN8 expression affects function of primary cancer cells *in vitro*.

(a) Histograms show the mean numbers of spheres formed by primary cancer cells with or without *TSPAN8* or *ATXN3* shRNA expression. Experiments were repeated three times independently. A total of 3 independent patients were tested. Two-tailed unpaired Student's T-test was performed. $^{**}P < 0.01$ and $^{***}P < 0.001$.

(b) Primary cancer cells with or without *TSPAN8* or *ATXN3* shRNA expression were treated with ADR at the indicated concentrations for 24 h. Experiments were repeated three times independently. A total of 3 independent patients were tested. The cell viabilities were determined. Two-tailed unpaired Student's T-test was performed. $^{**}P < 0.01$ and $^{***}P < 0.001$.

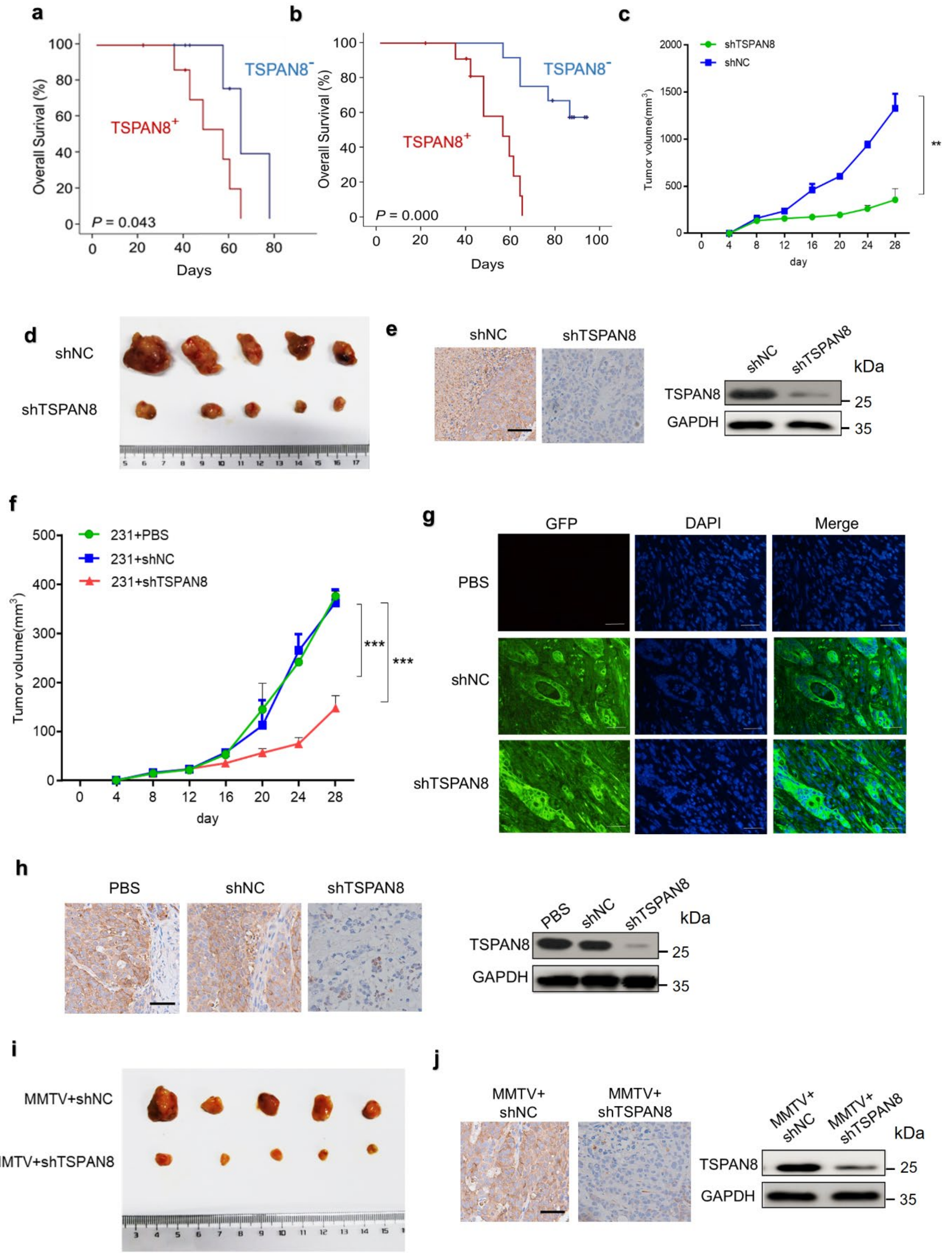

Supplementary Figure 6. TSPAN8 promotes tumorigenesis *in vivo*.

(a, b) Flow cytometry was used to separate $TSPAN8^+$ from $TSPAN8^-$ cells derived from human breast cancer patients. Kaplan-Meier survival analysis was performed of those mice with transplantation. The survival time of the mice with tumor formation (a, $P = 0.043$, N = 18 mice) and all mice with and without tumor formation (b, $P = 0.000$, N = 24 mice) was calculated (generated using 10^6 cells injection). *P* value was calculated by log-rank test.

(c-e) Breast cancer cells from three different patients were injected into mice. For each experiment, primary breast cancer cells taken from one patient were transfected with either control shRNA (shNC) or shRNA directed against TSPAN8 (shTSPAN8). For each experiment, $1x10^5$ cells were injected into NOD/SCID mice mammary fat pads. Tumor volumes were measured every four days. 5 animals were injected in each experimental group and the results shown are from one representative experiment using cells from one patient. (c). Each bar represents the mean ± SD. Tumor removed one month after injection were shown (d). The expression levels of TSPAN8 in these tumors were examined by IHC staining (scale bar = 100 mm) and immunoblotting analyses (e). $^{**}P < 0.01$.

(f-h) NOD/SCID mice were injected with $1x10^7$ MDA-MB-231cells. Multiple-point intratumoral injection of lentivirus expressing *TSPAN8*-shRNA and shNC was performed every three days. PBS injection was used in a blank control group. Tumor volumes were measured every four days (f). N = 5 each group. Each bar represents the mean ± SD. $^{***}P < 0.001$, 2-way ANOVA. The infection efficiency reached about 90%, which was determined by lentivirus-expressed GFP in tumor tissues (scale bar = 50 mm) (g). The expression levels

of TSPAN8 in these tumors were examined by IHC (scale bar = 100 mm) and immunoblotting analyses (h).

(i, j) Tumors removed from PyMT-MMTV mice after injection with lentivirus expressing *TSPAN8*-shRNA and shNC are shown (N = 5 each group) (i). The expression levels of TSPAN8 in these tumors were examined by IHC (scale bar = 100 mm) and immunoblotting analyses (j).

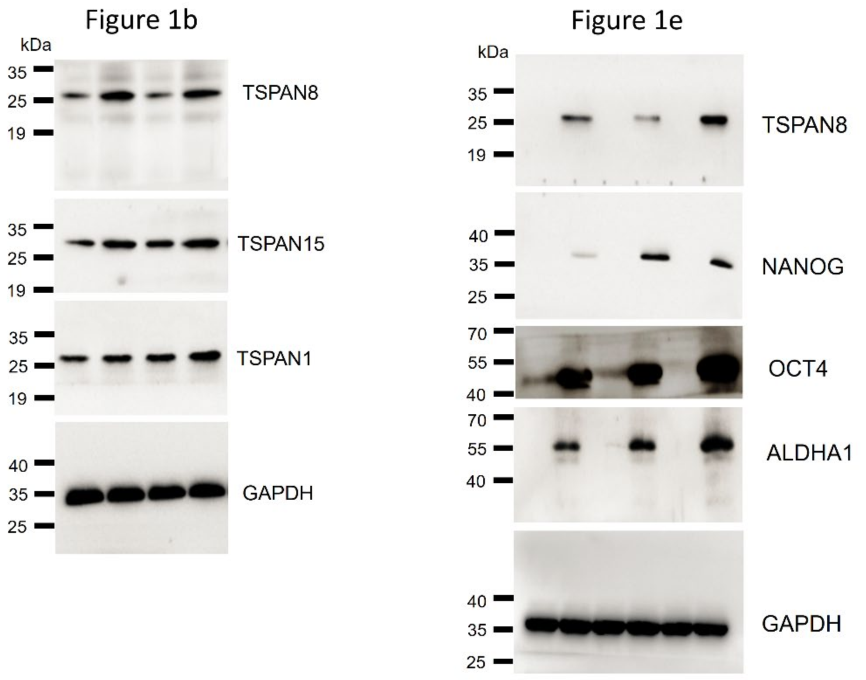

Supplementary Figure 7. Uncropped images of immunoblots for Figure 1b, 1e.

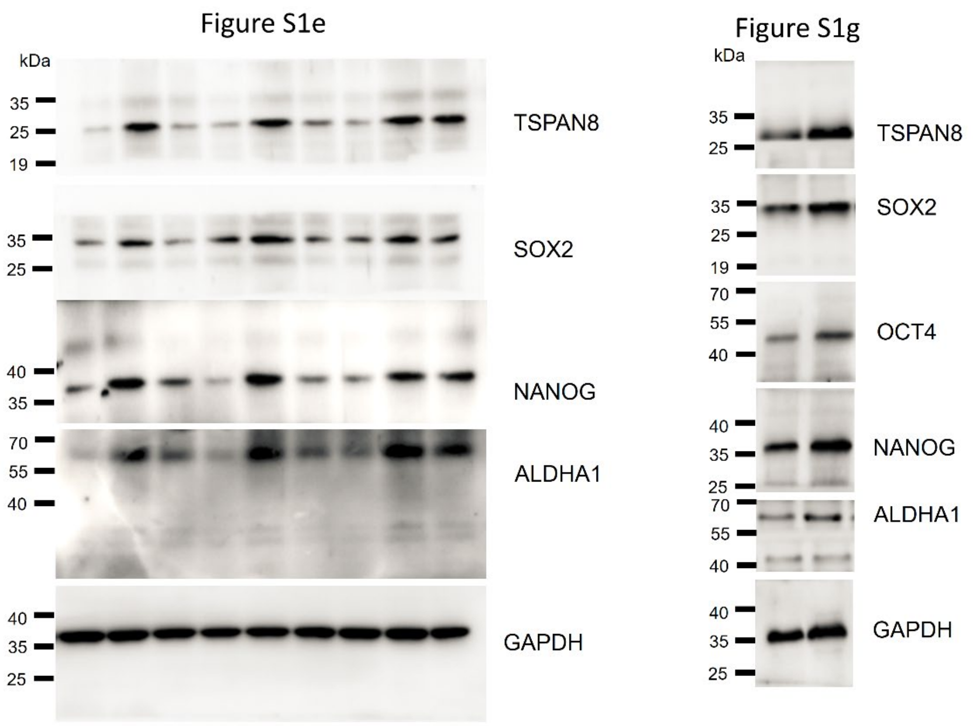

Supplementary Figure 8. Uncropped images of immunoblots for Figure S1e, S1g.

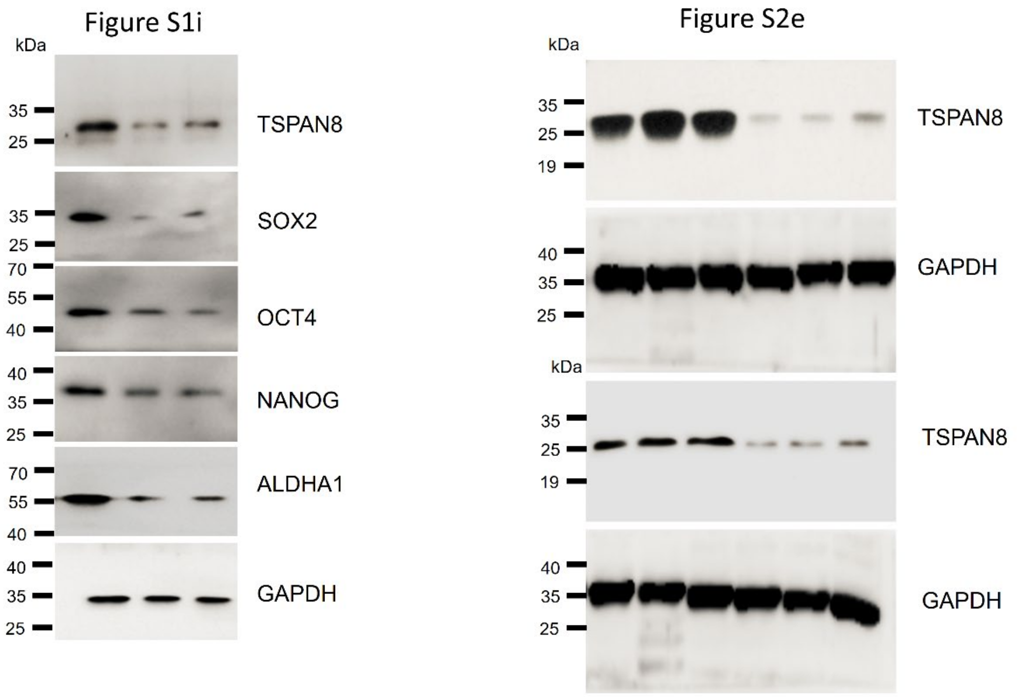

Supplementary Figure 9. Uncropped images of immunoblots for Figure S1i, S2e.

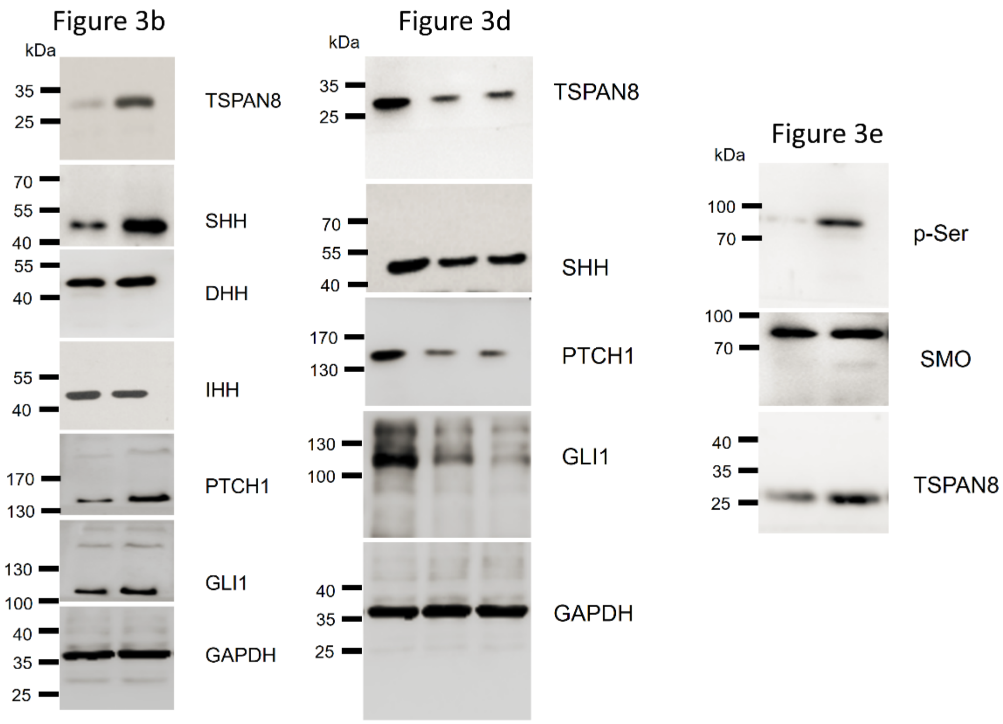

Supplementary Figure 10. Uncropped images of immunoblots for Figure 3b-e.

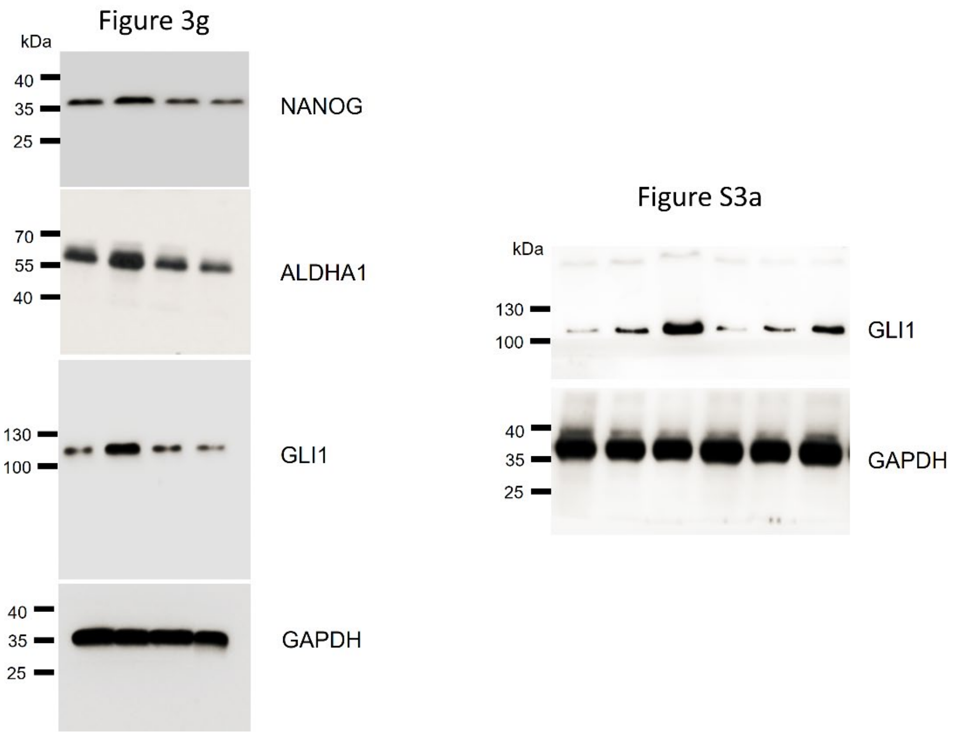

Supplementary Figure 11. Uncropped images of immunoblots for Figure 3g, S3a.

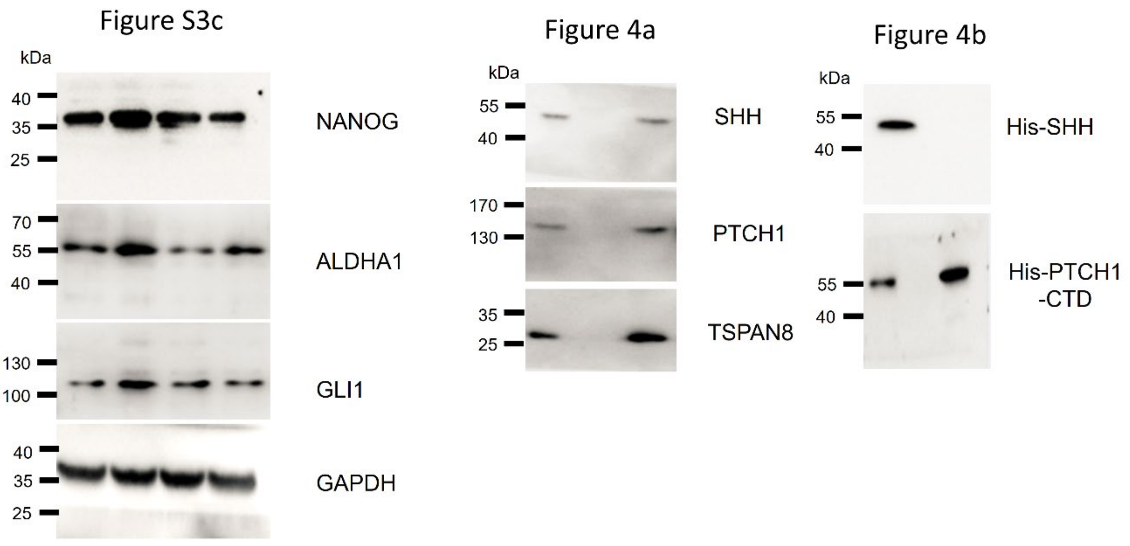

Supplementary Figure 12. Uncropped images of immunoblots for Figure S3c, 4a, 4b.

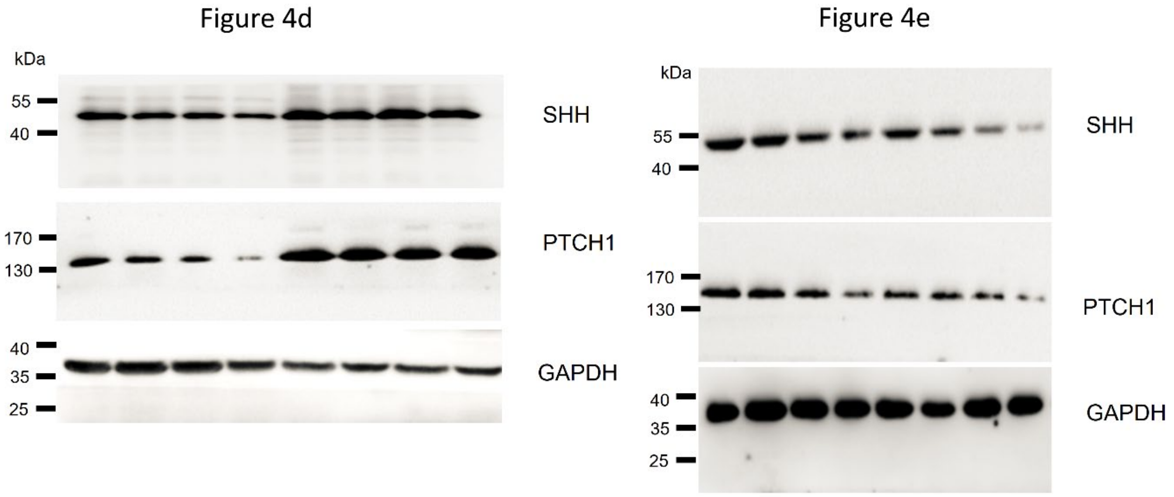

Supplementary Figure 13. Uncropped images of immunoblots for Figure 4d, 4e.

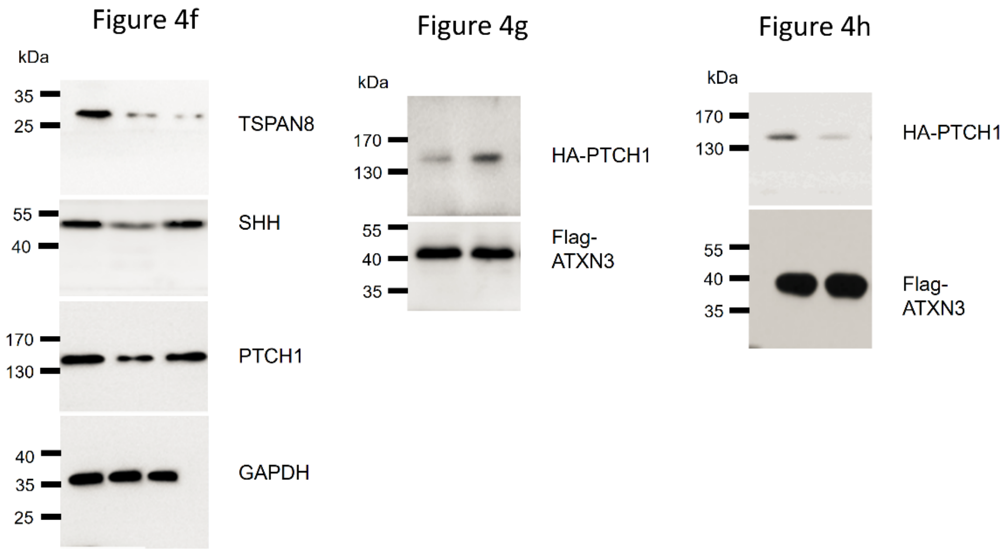

Supplementary Figure 14. Uncropped images of immunoblots for Figure 4f-h.

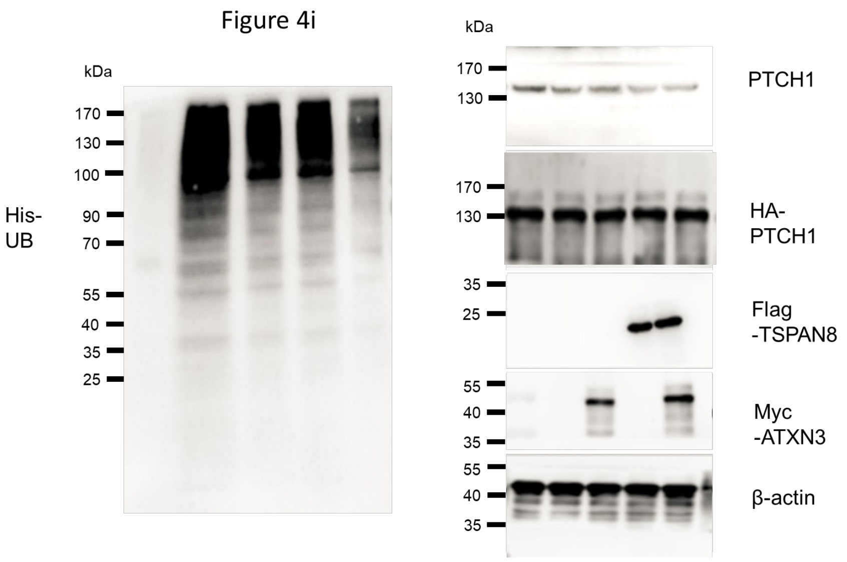

Supplementary Figure 15. Uncropped images of immunoblots for Figure 4i.

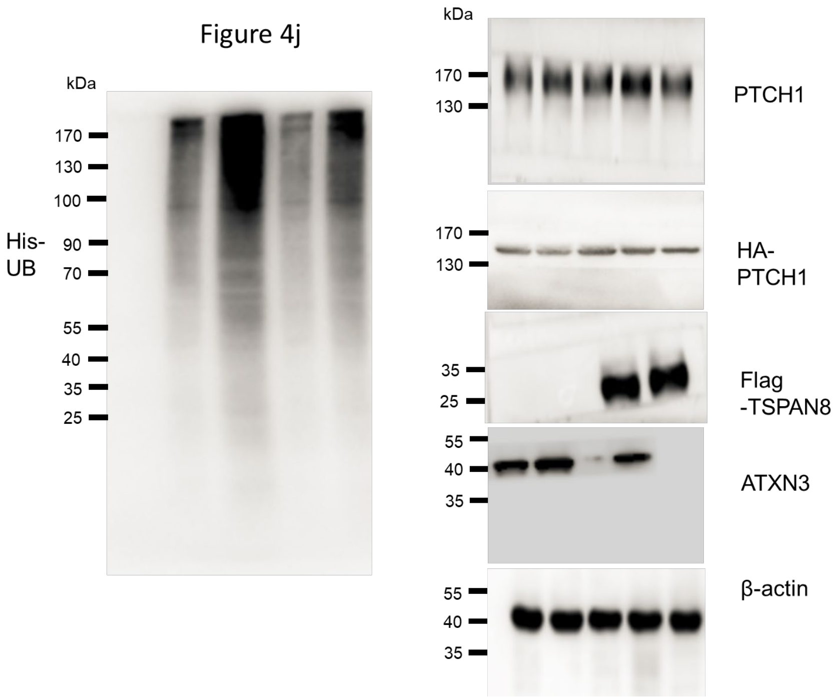

Supplementary Figure 16. Uncropped images of immunoblots for Figure 4j.

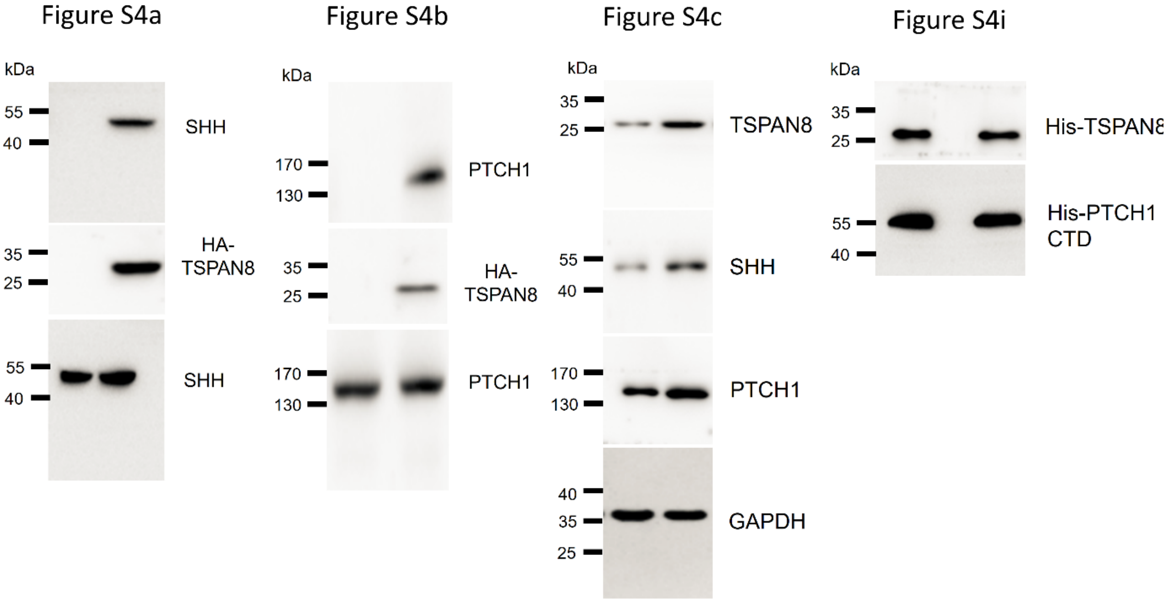

Supplementary Figure 17. Uncropped images of immunoblots for Figure S4a-c, S4i.

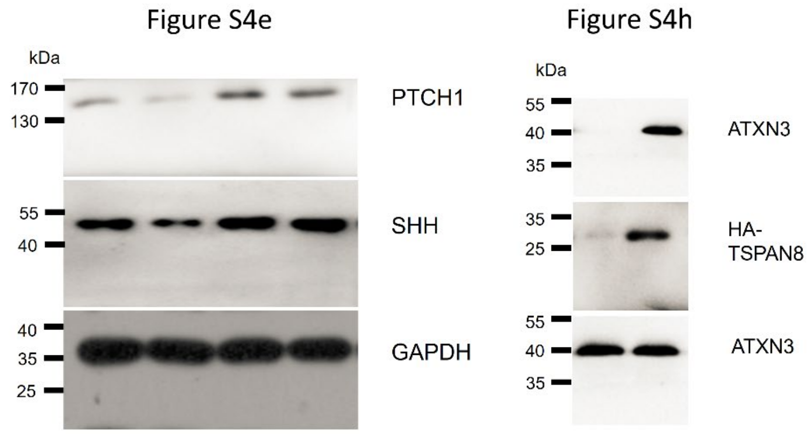

Supplementary Figure 18. Uncropped images of immunoblots for Figure S4e, S4h.

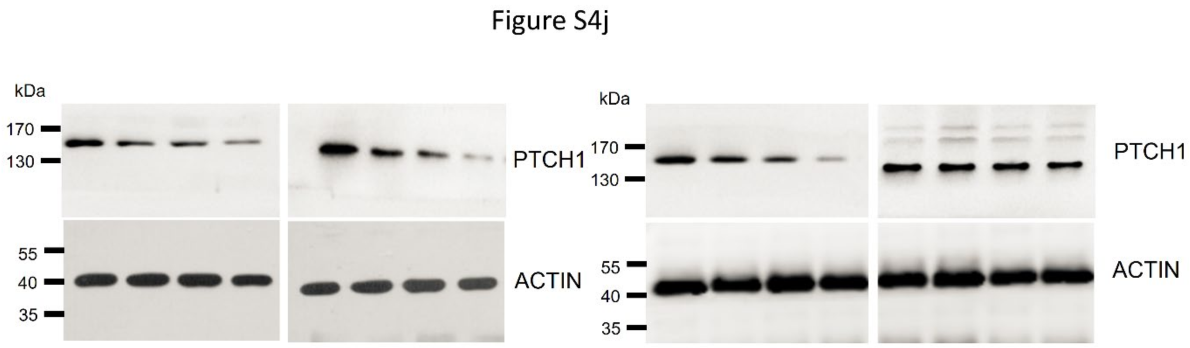

Supplementary Figure 19. Uncropped images of immunoblots for Figure S4j.

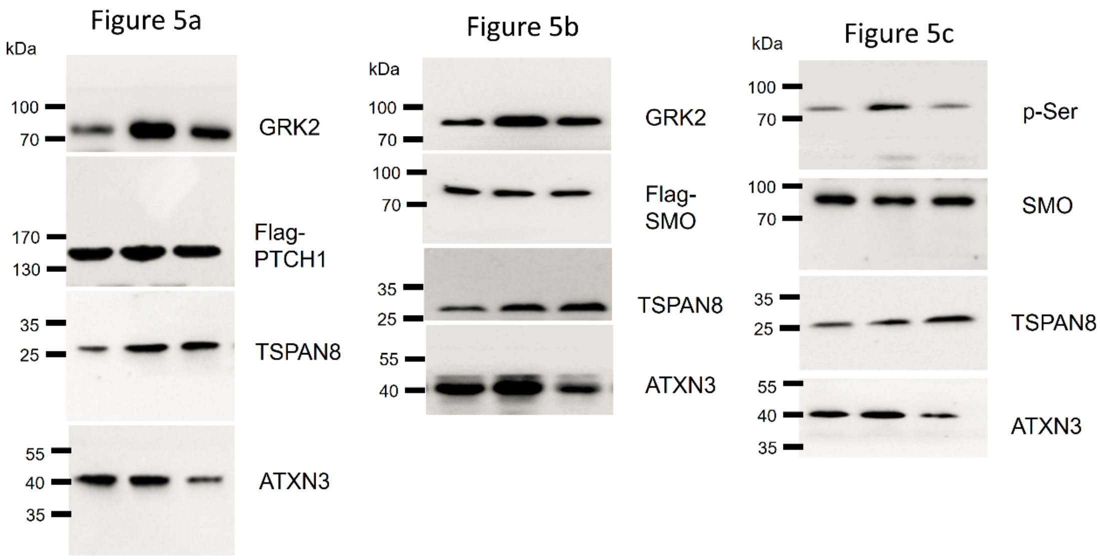

Supplementary Figure 20. Uncropped images of immunoblots for Figure S5a-c.

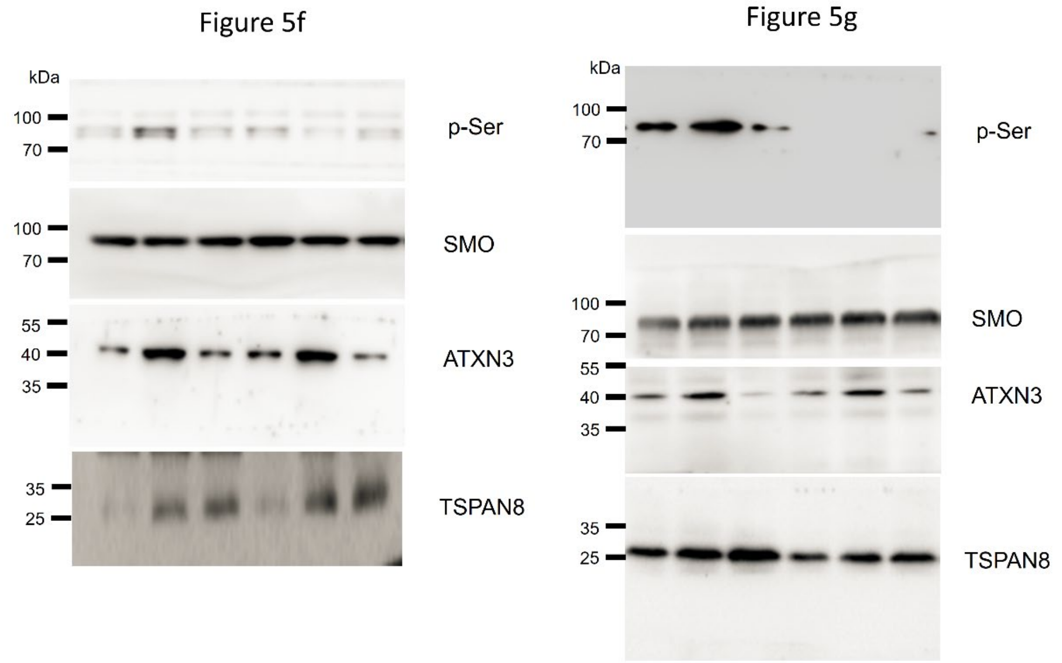

Supplementary Figure 21. Uncropped images of immunoblots for Figure S5f, 5g.

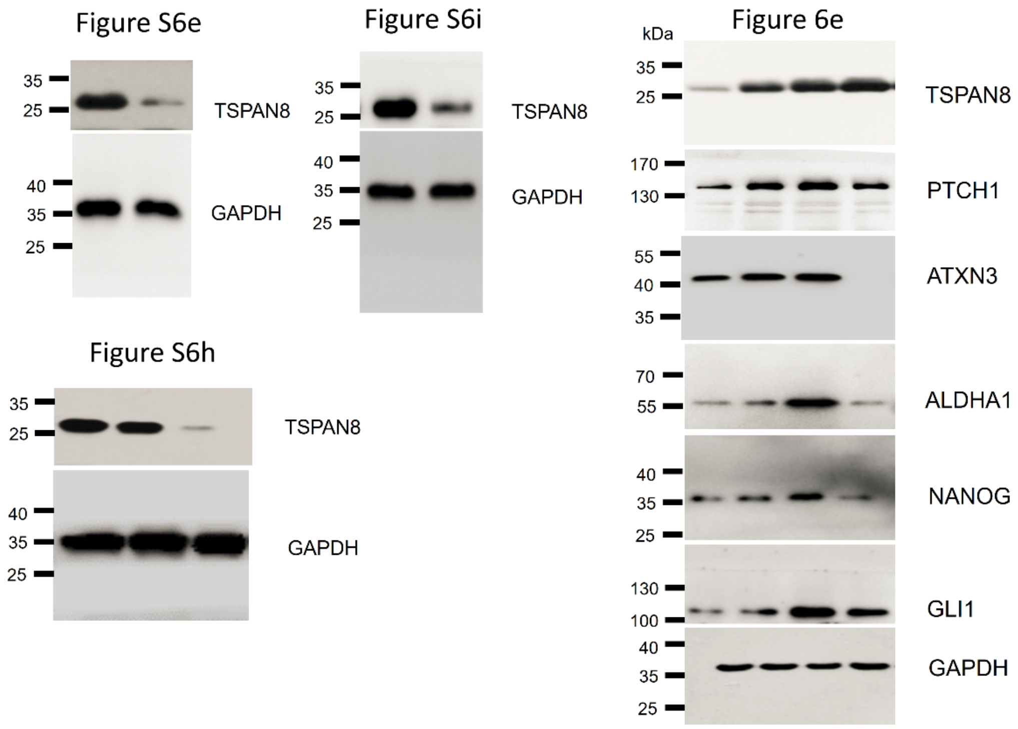

Supplementary Figure 22. Uncropped images of immunoblots for Figure S6e, S6i, S6h, 6e.

Supplementary Table 1. siRNA sequences used in our research.

siRNA	Sense (5'-3')	Anti-sense (5'-3')
siRNA-TSPAN8-1	GUAUCUUGAUCCUAGCAUU	AAUGCUAGGAUCAAGAUAC
siRNA-TSPAN8-2	GUCUGAUCGCAUUGUGAAU	AUUCACAAUGCGAUCAGAC
siRNA-NC (Negative Control)	UUCUCCGAACGUGUCACGUTT	ACGUGACACGUUCGGAGAATT

Supplementary Table 2. Correlation between TSPAN8 staining level and clinico-pathological parameters of patients with BCC.

Variable	Number	TSPAN8 staining		
		Low	Moderate / High	**p-value**
**Age (years)**				
< 50	32	9	23	
≥ 50	58	13	45	**0.546**
**Tumor size (cm)**				
≥ 1	37	12	25	
< 1	53	29	24	**0.036***
**Histologic grade**				
I - II	56	19	37	
III	34	4	30	**0.037***
**TNM stage**				
I - II	48	23	25	
III - IV	42	5	37	**0.000***

Supplementary Table 3. shRNA sequences used in our research.

shRNA	Sense (5'-3')	Anti-sense (5'-3')
shRNA-TSPAN8#1	5'CCGG CATCAACCTATCGTCAGTCAA CTCGAG TTGACTGACGATAGGTTGATG TTTTTG 3'	5' AATTCAAAAA CATCAACCTATCGTCAGTCAA CTCGAG TTGACTGACGATAGGTTGATG 3'
shRNA-TSPAN8#2	5'CCGG TGATCGCATTGTGAATGAAAC CTCGAG GTTTCATTCACAATGCGATCA TTTTTG 3'	5' AATTCAAAAA TGATCGCATTGTGAATGAAAC CTCGAG GTTTCATTCACAATGCGATCA 3'
shRNA-TSPAN8#3	5'CCGG CATTTGGACTGGCAGTTATTG CTCGAG CAATAACTGCCAGTCCAAATG TTTTTG 3'	5' AATTCAAAAA CATTTGGACTGGCAGTTATTG CTCGAG CAATAACTGCCAGTCCAAATG 3'

Supplementary Table 4. The primers used for real-time PCR in our research.

Gene	Sense (5'-3')	Anti-sense (5'-3')
TSPAN8	TGCCTGGAGATAGCCTTTGC	ACCACATAGCCAGAACAAGAAG
NANOG	ACAACTGGCCGAAGAATAGCA	GGTTCCCAGTCGGGTTCAC
ALDHA1	TGTTAGCTGATGCCGACTTG	TTCTTAGCCCGCTCAACACT
HHIP	GGGCGCCTGGAGAATAAGATAT	GTGGAGAGCAAAGTGCACATTTG
HHAT	GGACTCGGAAGTGCCGAAAG	CTCCTCTTCGTGTTCTCTGGA
SHH	AGAGGAGGCACCCCAAAAAG	TACACCTCTGAGTCATCAGCCT
DHH	GTGCCGCTACTCTACAAGCA	TACAACGCTCGGTCATCAGG
IHH	TCCGTCAAGTCCGAGCAC	CTCGATGACCTGGAAGGCTC
PTCH1	CCCCTGTACGAAGTGGACACTC	AAGGAAGATCACCACTACCTTGG
GLI1	CCTCTGAGACGCCATGTTCA	GAAAAGAGTGGGCCCTCGG
HES1	TACTTCCCCAGCACACTTGG	CGGACATTCTGGAAATGACA
HES2	GGCACTCTCGGAATCCTATG	TTTGAAGATGCTTCAGGCAA
HEY1	CGAAATCCCAAACTCCGATA	TGGATCACCTGAAAATGCTG
CYCLIND	CCGTCCATGCGGAAGATC	GAAGACCTCCTCCTCGCACT
LEF1	CTGCTAGAGACGCTGATCCA	TGGCTCTTGCAGTAGACGAA
AXIN2	TCACCAAACCCATGTCTGTC	TCCAGGAAAGTTCGGAACAG
