# Supplementary material for: TSPAN8 promotes cancer cell stemness via activation of sonic Hedgehog signaling
Source: Nat Commun. 2019 Jun 28;10:2863. doi: 10.1038/s41467-019-10739-3 (PMC6599078; doi:10.1038/s41467-019-10739-3)
Supplement: Supplementary file 2 — Reporting Summary [file 41467_2019_10739_MOESM2_ESM.pdf]

nature research

Corresponding author(s): Hongxia Wang

Last updated by author(s): Apr 13, 2019

Reporting Summary

Nature Research wishes to improve the reproducibility of the work that we publish. This form provides structure for consistency and transparency in reporting. For further information on Nature Research policies, see Authors & Referees and the Editorial Policy Checklist.

Statistics

For all statistical analyses, confirm that the following items are present in the figure legend, table legend, main text, or Methods section.

n/a	Confirmed	
☐	☒	The exact sample size (*n*) for each experimental group/condition, given as a discrete number and unit of measurement
☐	☒	A statement on whether measurements were taken from distinct samples or whether the same sample was measured repeatedly
☐	☒	The statistical test(s) used AND whether they are one- or two-sided<br>*Only common tests should be described solely by name; describe more complex techniques in the Methods section.*
☐	☒	A description of all covariates tested
☐	☒	A description of any assumptions or corrections, such as tests of normality and adjustment for multiple comparisons
☐	☒	A full description of the statistical parameters including central tendency (e.g. means) or other basic estimates (e.g. regression coefficient) AND variation (e.g. standard deviation) or associated estimates of uncertainty (e.g. confidence intervals)
☐	☒	For null hypothesis testing, the test statistic (e.g. *F*, *t*, *r*) with confidence intervals, effect sizes, degrees of freedom and *P* value noted<br>*Give P values as exact values whenever suitable.*
☐	☒	For Bayesian analysis, information on the choice of priors and Markov chain Monte Carlo settings
☐	☒	For hierarchical and complex designs, identification of the appropriate level for tests and full reporting of outcomes
☐	☒	Estimates of effect sizes (e.g. Cohen's *d*, Pearson's *r*), indicating how they were calculated

Our web collection on statistics for biologists contains articles on many of the points above.

Software and code

Policy information about availability of computer code

Data collection	/
Data analysis	/

For manuscripts utilizing custom algorithms or software that are central to the research but not yet described in published literature, software must be made available to editors/reviewers. We strongly encourage code deposition in a community repository (e.g. GitHub). See the Nature Research guidelines for submitting code & software for further information.

Data

Policy information about availability of data

All manuscripts must include a data availability statement. This statement should provide the following information, where applicable:

- Accession codes, unique identifiers, or web links for publicly available datasets
- A list of figures that have associated raw data
- A description of any restrictions on data availability

The data that support the findings of this study are available within the article, its Supplementary Information files and from the corresponding author upon reasonable request.

Field-specific reporting

Please select the one below that is the best fit for your research. If you are not sure, read the appropriate sections before making your selection.

☒ Life sciences ☐ Behavioural & social sciences ☐ Ecological, evolutionary & environmental sciences

Life sciences study design

All studies must disclose on these points even when the disclosure is negative.

Sample size	All data were statistically analyzed with GraphPad Prism 6.0 and SPSS 20.0 software. Two-tailed t-test was utilized to analyze the difference between the two groups. Pearson's test was applied to determine the correlation between clinicopathological parameters and protein expression. Data were presented as mean ± SD or SEM. Differences at P < 0.05 were considered statistically significant.
Data exclusions	There was no any inclusion/exclusion criteria.
Replication	Data represent the mean ± SD of three times of independent experiments. *P < 0.05, **P < 0.01 and ***P < 0.001.
Randomization	For the genetic therapeutic approach, 15 tumor-bearing nude mice were randomly divided into 3 groups, 5 in each group for intratumoral injection. The injection was performed by intratumoral multiple-point injection every 3 days.
Blinding	The TSPAN8 expression in tissues was scored blindly and independently by two scientists.

Reporting for specific materials, systems and methods

We require information from authors about some types of materials, experimental systems and methods used in many studies. Here, indicate whether each material, system or method listed is relevant to your study. If you are not sure if a list item applies to your research, read the appropriate section before selecting a response.

Materials & experimental systems

n/a	Involved in the study
☐	☒ Antibodies
☐	☒ Eukaryotic cell lines
☐	☐ Palaeontology
☐	☒ Animals and other organisms
☐	☐ Human research participants
☐	☒ Clinical data

Methods

n/a	Involved in the study
☐	☐ ChIP-seq
☐	☒ Flow cytometry
☐	☐ MRI-based neuroimaging

Antibodies

Antibodies used	Antibodies for the human antigens CD45 (Alexa-450, eBioscience), EpCAM (PE, eBioscience), CK (FITC, eBioscience), CD24 (PE, eBioscience), CD44 (APC, eBioscience) and TSPAN8 (FITC, eBioscience) were used for fluorescence-activated cell sorting (FACS).
Validation	Antibodies are validated according to the manufacturer's website.

Eukaryotic cell lines

Policy information about cell lines

Cell line source(s)	Human breast cancer cell lines MDA-MB-231, HCC-1954, MCF-7, T47D, BT474, and MDA-MB-468 were obtained from ATCC and grown in RPMI 1640 medium (HyClone) supplemented with 1% penicillin-streptomycin and 10% fetal bovine serum at 37 °C with 5% CO2.
Authentication	All cell lines were authenticated by STR profiling and mycoplasma was tested every two weeks.
Mycoplasma contamination	All cell lines were authenticated by STR profiling and mycoplasma was tested every two weeks.
Commonly misidentified lines (See ICLAC register)	There were no commonly misidentified lines.

Palaeontology

Specimen provenance	The experiment with human tissues was approved by the Human Ethics Committee of Shanghai General Hospital, Shanghai Renji Hospital, and Shanghai Jiao Tong University School of Medicine (Shanghai, China).
Specimen deposition	The experiment with human tissues was approved by the Human Ethics Committee of Shanghai General Hospital, Shanghai Renji Hospital, and Shanghai Jiao Tong University School of Medicine (Shanghai, China).
Dating methods	A piece of approximate 1 cm3 breast cancer tissue was removed during an operation and washed with DMEM/F12 (1: 1) to

Dating methods

remove the adipose tissue. Next, the tissue was cut into 1 mm3 pieces, followed by the addition of 100 U/mL III collagenase, 100 U/mL penicillin, 150 U/mL hyaluronidase and DMEM/F12 (1: 1) medium and incubation for 12–18 h at 37 °C. After centrifugation for 4 min at 80 g, the supernatant enriched in adipocytes and fibroblasts was removed. Trypsin was added to the pellet. After digestion for 10 min, the pellet was gently and repeatedly swirled, filtered through a sieve, and centrifuged.

☒ Tick this box to confirm that the raw and calibrated dates are available in the paper or in Supplementary Information.

Animals and other organisms

Policy information about studies involving animals; ARRIVE guidelines recommended for reporting animal research

Laboratory animals	All animal studies were conducted according to the guidelines provided by the Animal Ethics Committee of the Institute of Health Sciences. MDA-MB-231 cells were subcutaneously inoculated of 5-week-old female nude mice from Shanghai Laboratory Animal Center.
Wild animals	There were no wild animals.
Field-collected samples	There were no field collected samples.
Ethics oversight	All animal studies were conducted according to the guidelines provided by the Animal Ethics Committee of the Institute of Health Sciences. MDA-MB-231 cells were subcutaneously inoculated of 5-week-old female nude mice from Shanghai Laboratory Animal Center.

Note that full information on the approval of the study protocol must also be provided in the manuscript.

Human research participants

Policy information about studies involving human research participants

Population characteristics	There were no population characteristics.
Recruitment	/
Ethics oversight	/

Note that full information on the approval of the study protocol must also be provided in the manuscript.

Clinical data

Policy information about clinical studies

All manuscripts should comply with the ICMJE guidelines for publication of clinical research and a completed CONSORT checklist must be included with all submissions.

Clinical trial registration	There were no clinical trial.
Study protocol	/
Data collection	/
Outcomes	/

ChIP-seq

Data deposition

☐ Confirm that both raw and final processed data have been deposited in a public database such as GEO.

☐ Confirm that you have deposited or provided access to graph files (e.g. BED files) for the called peaks.

Data access links<br>*May remain private before publication.*	/
Files in database submission	/
Genome browser session<br>(e.g. UCSC)	/

Methodology

Replicates	/
Sequencing depth	/

Antibodies	/
Peak calling parameters	/
Data quality	/
Software	/

Flow Cytometry

Plots

Confirm that:

☒ The axis labels state the marker and fluorochrome used (e.g. CD4-FITC).

☐ The axis scales are clearly visible. Include numbers along axes only for bottom left plot of group (a 'group' is an analysis of identical markers).

☒ All plots are contour plots with outliers or pseudocolor plots.

☒ A numerical value for number of cells or percentage (with statistics) is provided.

Methodology

Sample preparation	Briefly, tissues were mechanically chopped and were then digested at 37°C for 45 min with collagenase-1. The resulting suspension was treated with DNAse at room temperature for 5 minutes, washed, and dissociated with trypsin/EDTA for 10 minutes at 37 ° C, and filtered through a 70 µm filter.
Instrument	Briefly, tissues were mechanically chopped and were then digested at 37°C for 45 min with collagenase-1. The resulting suspension was treated with DNAse at room temperature for 5 minutes, washed, and dissociated with trypsin/EDTA for 10 minutes at 37 ° C, and filtered through a 70 µm filter. In order to detect the proportion of cancer stem cells, CD44+/ CD24- flow cytometry analysis and FACS was performed using dual-staining for CD24 and CD44 with propidium iodide exclusion of non-viable cells. Trypsin-digested cells were washed and centrifuged for 5min at 1,200 rpm,1 µL of antibody was added to the samples, which were next shielded from light and left undisturbed for 15 min at 4 °C.
Software	Flow sorting was performed with a BD FACSAria II cell sorter (Becton Dickinson). The side scatter width versus side scatter region (SSC-W versus SSC-A) and the forward scatter width and forward scatter height (FSC-W versus FSC-H) were used to eliminate dead cells and cell clumps .
Cell population abundance	Then, we used TSPAN8 antibodies to sort TSPAN8-negative and TSPAN8-positive cells. In order to detect the proportion of cancer stem cells, CD44+/ CD24- flow cytometry analysis and FACS was performed using dual-staining for CD24 and CD44 with propidium iodide exclusion of non-viable cells.
Gating strategy	Flow sorting was performed with a BD FACSAria II cell sorter (Becton Dickinson). The side scatter width versus side scatter region (SSC-W versus SSC-A) and the forward scatter width and forward scatter height (FSC-W versus FSC-H) were used to eliminate dead cells and cell clumps .

☒ Tick this box to confirm that a figure exemplifying the gating strategy is provided in the Supplementary Information.

Magnetic resonance imaging

Experimental design

Design type	/
Design specifications	/
Behavioral performance measures	/

Acquisition

Imaging type(s)	/
Field strength	/
Sequence & imaging parameters	/
Area of acquisition	/

Diffusion MRI ☐ Used ☒ Not used

Preprocessing

Preprocessing software: /

Normalization: /

Normalization template: /

Noise and artifact removal: *Describe your procedure(s) for artifact and structured noise removal, specifying motion parameters, tissue signals and physiological signals (heart rate, respiration).*

Volume censoring: /

Statistical modeling & inference

Model type and settings: /

Effect(s) tested: /

Specify type of analysis: ☐ Whole brain ☐ ROI-based ☐ Both

Statistic type for inference (See Eklund et al. 2016): /

Correction: /

Models & analysis

n/a	Involved in the study
☐	☐ Functional and/or effective connectivity
☐	☐ Graph analysis
☐	☐ Multivariate modeling or predictive analysis

Functional and/or effective connectivity: /

Graph analysis: /

Multivariate modeling and predictive analysis: /
